# Impact of biodiversity loss on production in complex marine food webs mitigated by prey-release

**DOI:** 10.1038/ncomms7657

**Published:** 2015-03-23

**Authors:** Tak Fung, Keith D. Farnsworth, David G. Reid, Axel G. Rossberg

**Affiliations:** 1 National University of Singapore, Department of Biological Sciences, 14 Science Drive 4, Singapore 117543, Singapore; 2 Queen’s University Belfast, School of Biological Sciences, Belfast BT9 7BL, UK; 3 Fisheries Science Services, Marine Institute, Rinville, Oranmore, County Galway, Ireland; 4 Centre for Environment, Fisheries and Aquaculture Science (Cefas), Suffolk NR33 0HT, UK

## Abstract

Public concern over biodiversity loss is often rationalized as a threat to ecosystem functioning, but biodiversity-ecosystem functioning (BEF) relations are hard to empirically quantify at large scales. We use a realistic marine food-web model, resolving species over five trophic levels, to study how total fish production changes with species richness. This complex model predicts that BEF relations, on average, follow simple Michaelis–Menten curves when species are randomly deleted. These are shaped mainly by release of fish from predation, rather than the release from competition expected from simpler communities. Ordering species deletions by decreasing body mass or trophic level, representing ‘fishing down the food web’, accentuates prey-release effects and results in unimodal relationships. In contrast, simultaneous unselective harvesting diminishes these effects and produces an almost linear BEF relation, with maximum multispecies fisheries yield at ≈40% of initial species richness. These findings have important implications for the valuation of marine biodiversity.

## Abstract (web summary)

Biodiversity-ecosystem functioning relationships are not well characterized for large marine ecosystems. Using a dynamic model of complex marine food webs, Fung *et al*. find that release of fish from predation, not competition, is the principal mechanism shaping this relationship.

## Graphical abstract

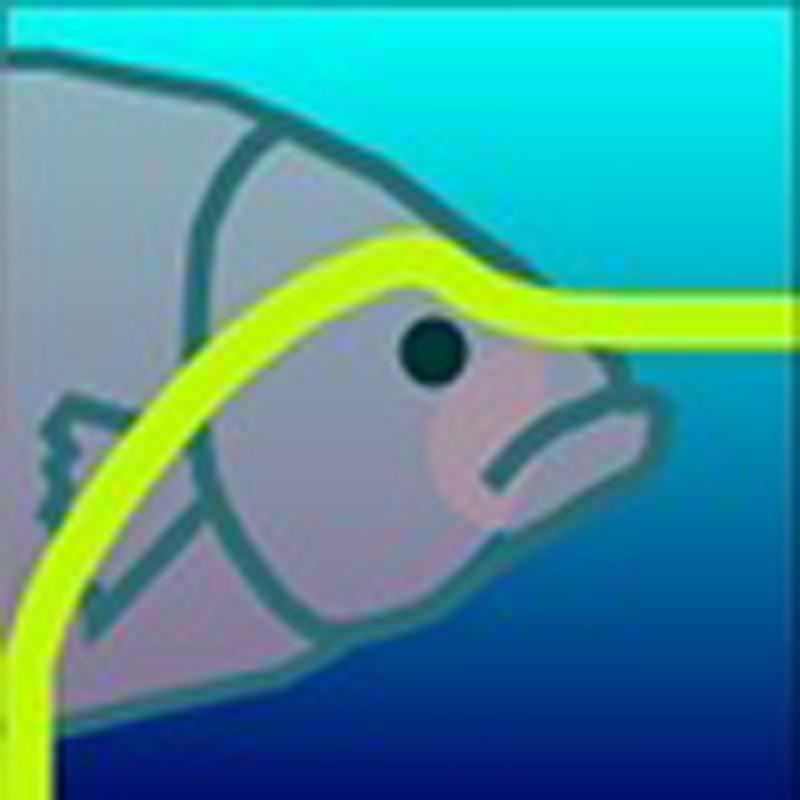

Biodiversity-ecosystem functioning (BEF) relations have been studied empirically^1^,^2^,^3^,^4^,^5^,^6^,^7^ and theoretically^8^,^9^,^10^,^11^,^12^, yet our understanding of these for large marine ecosystems (LME) remains vague^13^. Direct experimental studies in large ecosystems are prohibitive and the interpretation of comparative analyses in this context, including the problem of controlling for confounding variables, is an issue of ongoing debate^4^,^6^,^14^,^15^,^16^,^17^. On the other hand, simulation studies have so far been constrained to small, simple systems that have fewer than 100 species or two trophic levels^8^,^9^,^11^,^12^,^17^, leaving unanswered the question of how results could be scaled up, for example to LME.

To overcome these limitations, we use an innovative marine food-web model that resolves thousands of species over five trophic levels to study how total fish production (the rate of production of biomass by all fish species) is expected to change with fish species richness, a commonly studied BEF relation with important practical applications. In particular, the model incorporates omnivory, which is ubiquitous in marine ecosystems^18^,^19^,^20^, but hitherto neglected by food-web models used in BEF studies^21^. This feature permits the emergence of complex network topologies, thus building on previous modelling studies that use layered food webs with no omnivory and discrete trophic levels^21^. Species were first deleted at random from model food webs one-by-one, allowing the effects of species composition to be controlled by averaging over replicate random sequences^22^,^23^. Random deletions correspond to the case where no species traits affect the probability of extinction, which is an abstraction in view of empirical evidence for non-random species loss^24^,^25^,^26^. Therefore, we also quantify the relationship between total fish production and fish species richness using deletions in order of (a) decreasing body mass, (b) decreasing trophic level and (c) decreasing species population biomass. These correspond to the observed fisheries practice of targeting fish species with large body masses^27^,^28^, high trophic levels^29^,^30^ and large biomasses^31^,^32^, respectively. Furthermore, we also examine deletions in order of (d) decreasing connectivity (number of trophic links), to test the hypothesis that the most connected species are the most important for ecological functioning^33^.

Using our model, we show that a realistically complex food web is nevertheless expected to produce a simple BEF curve under random deletion of species, with the average trend following a Michaelis–Menten function. We find that release of fish from predation is the main mechanism shaping BEF relations, in contrast to previous expectations^31^ that various forms of competition would dominate, as in simpler communities^8^. Effects of interactions between the deleted species and other species separated by at least two trophic links—that is, indirect interactions—largely cancel, resulting in a net effect weaker than the direct interactions. Furthermore, we find that deletions in order of decreasing body mass or trophic level amplify prey-release, leading to greater gains in production following species loss. Conversely, deletions in order of decreasing biomass resulted in convex (upward-bending) BEF relations, representing severe declines in ecosystem functioning even with loss of relatively few fish species. Deletions in order of decreasing connectivity resulted in almost linear BEF relations, thus providing partial support for the hypothesis that removal of the most connected species has the biggest impact on functioning^33^.

Our quantitative predictions of how marine fish production depends on species richness fill a key knowledge gap in biodiversity research and ecosystem management. Importantly, our findings provide a mechanistic understanding of situations where biodiversity loss can lead to gains in ecosystem functioning. As such, they refine our understanding of the generality of loss of provisioning ecosystem services as a main argument for biodiversity conservation^22^.

## Results

### Generation of model food webs and their validation

The Population-Dynamical Matching Model^34^ (PDMM; see Methods, Supplementary Methods, Supplementary Fig. 1, Supplementary Table 1) simulates population dynamics in food webs linking thousands of species. It is used here because it is the only model capable of generating sufficiently complex food webs that realistically represent those in LME^35^. The PDMM is founded on well-understood theory^36^ and earlier applications have demonstrated its quantitative strengths in describing marine community structure and dynamics, in particular at higher trophic levels^35^,^37^,^38^. Ecological model communities are generated by the PDMM via an assembly algorithm that iteratively introduces random variant species into a food web. Assembly is considered complete when species richness no longer increases on average as new species are introduced: a condition of saturation in which speciation is balanced by extinction. In our parameterization (Supplementary Methods), communities typically reached this point with around 4,000 coexisting species, of which around 150–300 were fish (taken to be all species with maturation body mass above 10^−3 ^kg; see Supplementary Methods for details).

We generated 20 model food webs from 20 independent runs of the PDMM assembly algorithm. These were verified by comparison with empirical data from large marine shelf communities, representing 10 key ecological properties (Table 1, Supplementary Methods, Supplementary Figs 2–5, Supplementary Tables 2–6). These properties cover biodiversity patterns, size structure and trophic structure.

### BEF relations under random species deletions

Simulated BEF relations were obtained from each of the 20 PDMM food webs by sequentially deleting randomly chosen fish species, with simulation of population dynamics of the diminished food web after each deletion until a dynamic equilibrium was reached. Biomass production summed over all fish species, *P*, was used as a measure of ecosystem functioning. Biodiversity of a food web was quantified by fish species richness expressed as a proportion *Φ* of the initial number of fish species.

**Figure 1:**
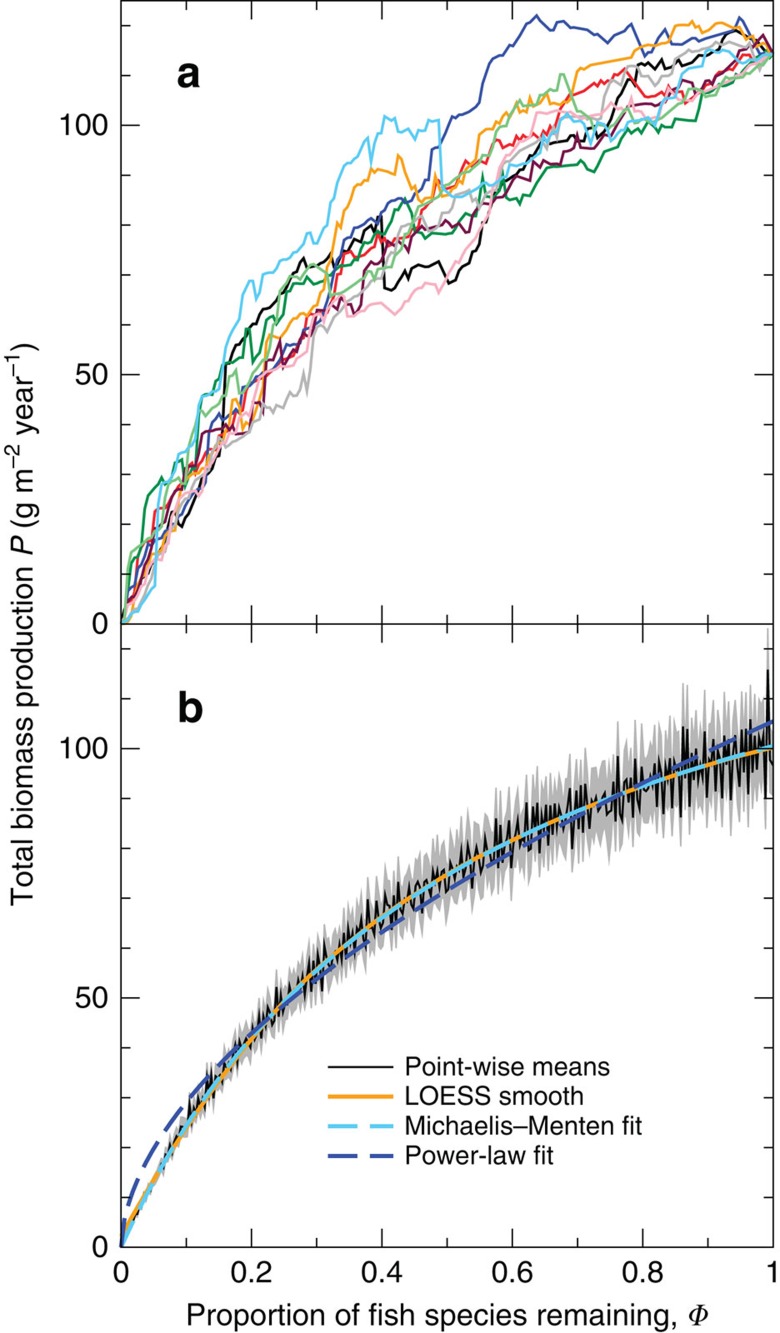
Predicted total fish biomass production against normalized fish species richness for random deletions. (**a**) Ten sample random deletion sequences for one model food web; the different colours represent separate sequences. (**b**) Point-wise means (black), s.e. values (grey), LOESS smooth of the point-wise means (orange) and two fitted curves as indicated in the legend, based on the 200 random deletion sequences for all 20 food webs.

To sample the variety of possible responses, 10 random deletion sequences were evaluated for each of the 20 model food webs, to produce an ensemble of 200 simulated BEF relations (Fig. 1a, Supplementary Figs 6 and 7). Each random deletion sequence consists of repeatedly choosing a fish species randomly, deleting it and then simulating community dynamics to a new equilibrium; this is continued until no fish species remain. We found moderate variation in total fish biomass production *P* for each of 300 intervals of *Φ* evenly spaced between 0 and 1 (CV≥0.17, increasing as *Φ* declined; Supplementary Fig. 8). This confirms empirical studies suggesting a strong influence of community composition on BEF relations^39^,^40^. The variation was mainly due to differences among food webs (Supplementary Methods, Supplementary Fig. 9). Additional variation attributable to random sequences was relatively small and only dominated for low *Φ*<0.1 (Supplementary Fig. 9).

To reveal patterns beyond the idiosyncratic changes identified above, mean total fish production was computed across the simulations for each value of *Φ* and the resulting curves smoothed using LOESS (Methods; Fig. 1b, black and orange lines). This analysis showed that mean production declines with each species deleted and that this decline becomes steeper as fewer species remain in the community, that is, the BEF relation is concave. This is consistent with previous results using smaller systems^1^,^3^,^5^, suggesting some generality across scales. The model predicts that one-quarter of the initial species richness is sufficient to maintain half of the initial production (Fig. 1b), implying that, on average, initial biodiversity loss only has minor impacts on production. However, this proportion translates to an average of 47 fish species for the 20 food webs, which is far more than the few species often found to maintain half of functioning in small-scale experiments^3^. The grey region in Fig. 1b denotes the s.e. values for the mean production values from simulations, which is an appropriate measure of uncertainty in these average values. Supplementary Figure 10 instead shows the s.d. values, which measure the variation in production values from the means.

In addition, we tested how well two parsimonious curves, each given by two parameters, fitted the smoothed BEF relation (non-linear least-square fits). An excellent fit (Fig. 1b, light blue dashed line) was obtained with the saturating Michaelis–Menten (MM) functional form^3^,^5^ given by *P*=*AΦ*/(*Φ*+*B*), with *R*^2^>0.999 and a root mean square (r.m.s.) approximation error of only 0.35 g m^−2^ year^−1^ (with *A*=154 g m^−2^ year^−1^, *B*=0.533). A non-saturating power-law of the form *P*=*CΦ*^*D*^ gave a worse fit to the smoothed relationship (Fig. 1b, dark blue dashed line), with *R*^2^=0.987 and an r.m.s. error of 3.01 g m^−2^ year^−1^ (*C*=105 g m^−2^ year^−1^, *D*=0.559), which is an order of magnitude larger. This suggests that with hypothetical higher species richness the BEF relation would indeed saturate. This result confirms conclusions drawn previously from a meta-analysis of experiments using smaller, simpler systems^3^,^5^ and extends them to LME.

Theoretically, an MM curve has been derived analytically for conceptually simple community models since the 1970s^10^,^36^,^41^. In this study, we find that an analytical model that is much simpler than the complex PDMM is able to reproduce the MM BEF relation derived from the PDMM (Supplementary Methods). This result is unexpected, in particular, because the analytical model assumes linear (Holling type I) consumer functional responses (Supplementary Methods), whereas our more complex simulation model assumes non-linear, extended Holling type II consumer functional responses (Methods). In practice, the difference between the two functional response types could have been small because the average satiation level^42^ (which varies from 0 to 1) of all fish species in each of the 20 complex model food webs did not exceed 0.384, which could have constrained their type II functional responses mostly to the approximately linear portions. Knowledge that relations between richness and biomass production in LME tend to follow MM curves, and are therefore largely determined by only two parameters, will greatly facilitate prediction of the effects of ongoing large changes in biodiversity.

### Analysis of mechanisms underlying the MM curve

**Figure 2:**
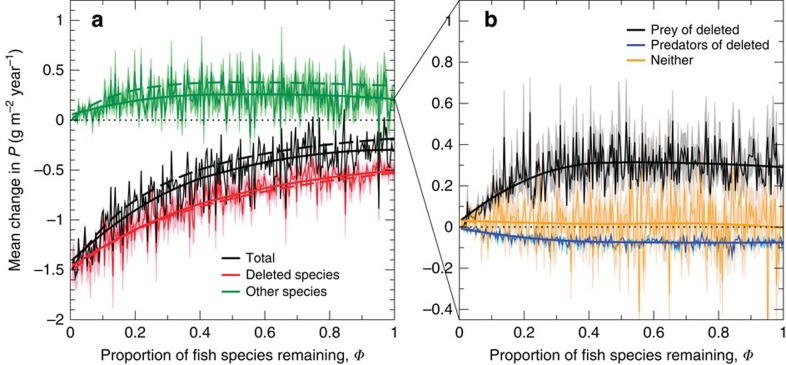
Components of total change in fish biomass production following random species deletions. Panel (**a**) splits the total change in production (*P*) into the production lost from the deleted species and responses from other species. In panel (**b**) the responses from other species are further split into those from prey and predators of the deleted species, as well as from species that were neither prey nor predators. Shown are point-wise means (thin lines) with s.e. values (pale colours) and LOESS smoothers (thick lines), based on the 200 random deletions for all 20 food webs. Dashed lines in panel (**a**) are analytic approximations.

The mean change in *P* resulting from the deletion of a randomly chosen species reflects the direct loss of production by that species plus the indirect response in production of the remaining species. If the direct loss was the only contribution, that is, if dynamic responses by other species did not affect *P* on average, then the mean BEF relation would necessarily be linear, because the mean direct effect is *P* divided by the number of extant species. The characteristic non-linear saturating form of the BEF relation is therefore entirely due to indirect effects, consistent with previous studies^8^,^9^,^12^. As a first step towards understanding the mechanisms underlying the shape of the BEF relation in our model, we separated the mean direct and indirect effects of the deletion of each species in the random deletion experiments (Fig. 2a). The figure also displays analytic approximations for the magnitudes of the direct and indirect contributions to the change in *P*, derived in Supplementary Methods from the MM form of the BEF relationship. The differences between simulations and these approximations result from occasional secondary extinctions of fish species (on average, one in four species deletions caused a secondary extinction; Supplementary Methods).

To further understand the driving mechanisms for the non-linear BEF relation, we resolve the indirect contribution into those from four categories of species, defined by their trophic relationship with the deleted species: (a) prey but not predator, (b) predator but not prey, (c) neither predator nor prey and (d) predator and prey. Contributions from the last category tended to be very small (Supplementary Fig. 11) and are not considered further. The total contributions from the three other categories, averaged over all 200 deletion sequences, are plotted in Fig. 2b against the proportion of species remaining, *Φ*. Interestingly, the average total contribution from prey of the deleted species tended to be much larger than that from those species that were neither prey nor predators (Fig. 2b). This is critically important: the latter category includes all those fish species that are mainly in a true or ‘apparent’ competitive relation with the deleted species. Competitive release therefore plays only a minor role in shaping the BEF relation in large complex food webs, despite its recognized importance for simpler communities^8^,^43^,^44^. The contribution from species that were predators of the deleted species was intermediate in magnitude between contributions from species that were prey of the deleted species and those that were neither prey nor predators (Fig. 2b). This contribution was negative and its smaller magnitude in comparison with the contribution from prey of the deleted species can be explained by inefficient transfer of energy from prey to predators. Previous modelling studies of marine communities have frequently demonstrated prey-release following depletion of predators, using EwE (Ecopath with Ecosim) and Atlantis^45^,^46^. However, these models did not fully resolve the communities to species level and also did not examine the consistency of this effect on BEF relations as species are sequentially deleted.

**Figure 3:**
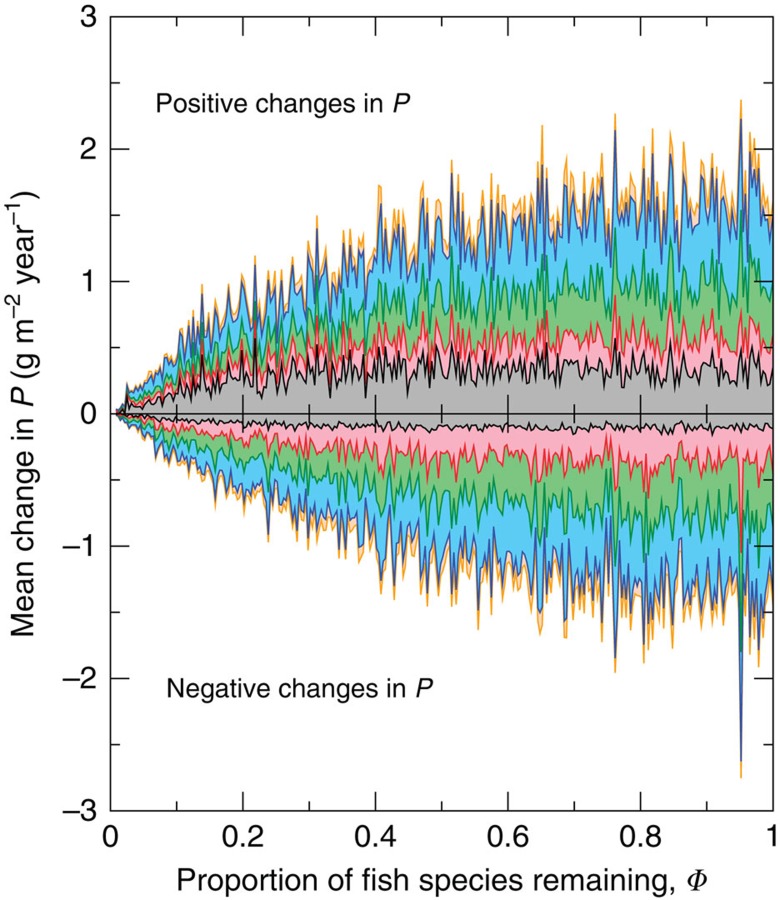
Contributions to mean total change in production by species with different minimum numbers of trophic links from deleted species, for random deletions. Considering all undeleted species with positive (above *x*-axis) and negative (below *x*-axis) changes in production following a random species deletion, the contributions to the mean total change in production from species that are a minimum of one (grey), two (pink), three (green), four (blue) and five (orange) trophic links away from the deleted species. Results are based on the 200 random deletions for all 20 food webs.

Decomposing the fish community’s response to species deletion even further, we show in Fig. 3 the sum of positive changes in production of fish species with different degrees of separation from the deleted species, as well as the sum of negative changes. Remarkably, species that were neither predators nor prey of the deleted species responded with larger positive and negative gross changes in production than prey and predators (Fig. 3). Contributions from fish species at four degrees of separation were largest, with a sharp decrease in contributions from species at higher degrees of separations. This could reflect more fish species with increasing degree of separation (each fish species is typically connected to many other species; Table 1), until nearly all fish species have been accounted for. The sum of the absolute positive and negative gross changes in production for species that were neither predator nor prey is typically at least an order of magnitude greater than the net change shown in Fig. 2b; the positive and negative changes mostly cancel each other.

### Effect of interaction asymmetry for BEF relations

Our random-deletion study demonstrates that while realistically complex food webs produce MM-shaped BEF curves as empirically found for single trophic systems^1^,^3^,^5^, the underlying mechanism is entirely different. For communities consisting of just one trophic level, the main structuring mechanisms are various forms of competition or their absence (niche differentiation)^44^, even when other interactions, for example facilitation by ecosystem engineers, also play a role^47^. Such competitive interactions are typically mediated through shared limiting resources, such as light, nutrients or food, and are therefore approximately symmetric. If competition is perfectly symmetric, then one can show mathematically (Supplementary Methods) that this leads to an increase in community production with each species added and a loss of production with each species deleted. Thus, the BEF relation is predictably positive in any instance. With approximate symmetry, one can expect the relation to be positive in the majority of instances.

When direct predator–prey interactions dominate in shaping BEF relations, as is the case here, this interaction symmetry is lost. Consequently, even though production declines for random deletion sequences on average, there are many instances where deletions lead to an increase in production—22% of deletions in our simulations (Fig. 1a, Supplementary Figs 6 and 7). In these complex food webs, a positive association between biodiversity and production is therefore not as inevitable as for competitive communities.

### Non-random deletion sequences

**Figure 4:**
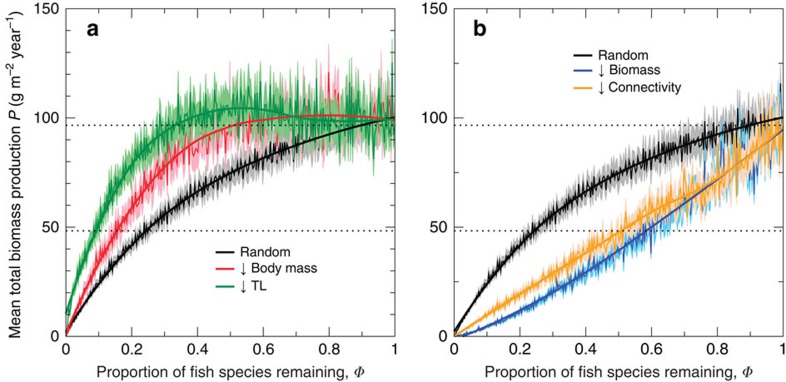
Predicted mean total fish biomass production against normalized fish species richness for ordered deletions. Production-richness relations are shown for (**a**) random deletions and deletions by decreasing body mass and trophic level (TL), and (**b**) random deletions and deletions by decreasing biomass and connectivity. For each relation, point-wise means (thin lines), s.e. values (pale colours) and LOESS smoothers (thick lines) are presented, based on the 20 ordered deletions for all 20 food webs. The two dotted horizontal lines mark the initial total fish biomass production and 50% of this value.

Deletions in order of decreasing body mass or decreasing trophic level both resulted in an increasing average production trend at high richness levels, before average production started to decline (Fig. 4a). When fish species were deleted in order of decreasing maturation body mass, the contributions from prey of the deleted species are inflated relative to the null random-deletion case (Supplementary Fig. 12a). The same result was found when species were deleted in order of decreasing trophic level (Supplementary Fig. 12b). In contrast, deletions in order of decreasing biomass or connectivity led to average production declining more quickly relative to the null scenario, with a convex shape for the BEF relation in the former case (Fig. 4b).

### Unselective multispecies fishing

**Figure 5:**
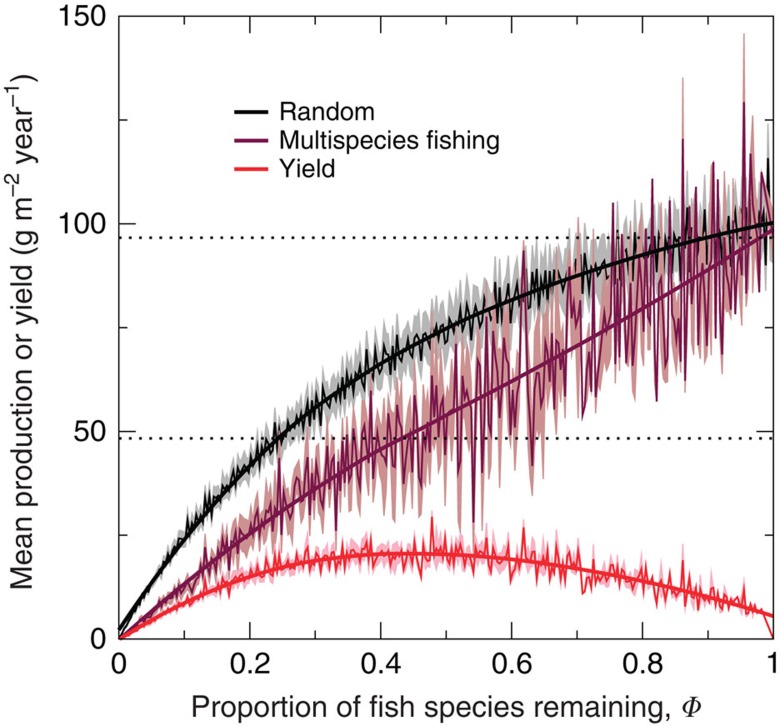
Predicted mean total fish biomass production and yield against normalized fish species richness for multispecies fishing. Production-richness relations are shown for random deletions and the case where all fish species are unselectively harvested at the same rate, with this rate increased from 0.06 to 8 year^−1^ in increments of 0.02 year^−1^. In addition, the relation between multispecies yield and richness is shown. For each relation, point-wise means (thin lines), s.e. values (pale colours) and LOESS smoothers (thick lines) are presented, based on results from all 20 food webs. The two dotted horizontal lines mark the initial total fish biomass production and 50% of this value.

In view of the strong dependence of BEF relations on the way in which species are deleted, the question arises as to what kind of relations will emerge for scenarios where fish species are harvested simultaneously rather than sequentially. We therefore also investigated the case of unselective multispecies fisheries, which has been studied in fisheries science^48^ and has been used to approximate fishing regimes for the North and Celtic Sea demersal fish communities^35^. Experiments were performed on each of the 20 model food webs where all fish species experienced a constant harvesting rate *H*, which varied in each experiment from 0.06 to 8 year^−1^ in increments of 0.02 year^−1^ (Methods). At *H*=8 year^−1^, no fish species survived in any of the 20 webs. The relation between *Φ* and mean total fish production in these fished webs is shown in Fig. 5, which follows a linear trend with declining biodiversity. We also include in Fig. 5 the mean values of fisheries yields corresponding to the fishing regimes applied (total fish biomass × *H*). Mean yield reaches the highest values at around *Φ*=0.4, where around 60% of fish species are extirpated.

## Discussion

Natural communities are generally thought of as complex systems with high interconnectedness of constituent components, yet previous models have fallen short of capturing the reticulate and adaptive nature of dynamic food webs in LME. Earlier mechanistic studies of BEF in food webs have focused on webs with a small number of species assigned to discrete trophic levels^9^,^11^,^12^. In such models, low trophic complementarity, that is, high overlap between species in their roles as consumers and resources, leads to high resource- and consumer-mediated competition and saturating or even hump-shaped BEF relationships^12^. For these simple discrete trophic level (layered) models, effective competitive (or ‘trophic niche’) overlaps as defined by Bastolla *et al*.^49^ and Chesson & Kuang^50^ are always positive, leading to a consistently negative effect of competition on abundance and production. However, in realistically complex non-layered food webs with many species and omnivory, appropriately defined effective competitive overlaps can be either positive or negative^36^. This explains the incoherent responses of indirectly connected species following random species deletions, which we report here (Figs 2b and 3). As a result, competition plays a much smaller role in determining BEF relations than direct predator–prey interactions. Furthermore, because a predator–prey pair has fundamentally asymmetric trophic effects on each other, production decreases only on average with each deletion of a random species, not in each instance as symmetric competitive models suggest (Supplementary Methods). In future work, there is a need to quantify the degree of symmetry in real competitive systems, especially at larger scales, to test the appropriateness of symmetry assumptions in competition models.

We found that using ordered instead of random deletion sequences qualitatively changed the shapes of the BEF relations (Fig. 4). This is consistent with results using simpler food-web models^9^,^51^, but our results are valuable in specifying how the BEF relations are expected to change in LME, which is *a priori* unclear due to their greater complexity. Deletions by decreasing maturation body mass or trophic level increased the effects of prey-release (Supplementary Fig. 12). This was because species with a larger maturation body mass or trophic level were generally able to feed on more species, representing a greater range of body masses achieved during growth, which increases the size range of prey that can be consumed. In contrast, deletions by decreasing biomass or connectivity both led to a steeper decline in production relative to random deletions. The underlying reason is that species with higher biomasses or that are more connected also tend to have higher production (Supplementary Fig. 13), such that species with high production tend to be removed first in both scenarios. This resulted in a sharply increasing, convex BEF relation (up to the pristine biodiversity) for deletions in order of decreasing biomass. A similar pattern has been found in observational studies of pollination by bee species^52^, dung burial by dung beetles^52^, biomass of coral reef fish species^6^,^17^ and biomass of deep-sea nematodes^16^,^17^. The cause of the sharply increasing, convex deep-sea nematode biomass trend has been postulated to be mutualistic interactions^16^; in contrast, the convex functioning trends found in the other three studies are more likely to be explained by the highest functioning species being the most extinction-prone^17^,^52^, such that species with the highest functioning are lost first—this is also how convex relationships between richness and production can be generated in our model food webs. In addition, our model results for deletions in order of decreasing connectivity are consistent with expectations from topological models^33^. Our results confirm that upward-bending BEF relations can arise when traits defining extinction risk and functioning overlap, using an explicitly mechanistic model. In this case, the loss in functioning dominates gains from prey-release (Supplementary Fig. 13), a finding that is *a priori* unclear and cannot simply be extrapolated from studies using simpler systems.

The time to reach a new equilibrium after a species deletion varied from 0.3 to 28,500 years in simulations, with a median of 22.5 years. In real marine ecosystems with heavy fishing pressure, there may be insufficient time in between species extinctions to allow the full effects of an extinction to be manifested. This could qualitatively alter the shapes of the BEF relations found^53^,^54^; for example, a saturating curve may become more linear due to weaker prey-release effects. In addition, we did not examine species invasions, which are common in coastal marine ecosystems^55^. Future studies could use the model food webs that we have generated to examine BEF relations under increasing species richness, representing species invasions.

We also found that when all fish species were simultaneously harvested in our model food webs, simulating the efforts of unselective multispecies fisheries, the BEF relation obtained was flatter than that in the random deletion null case (Fig. 5). Large species with low population growth rates typically became extinct first with increasing harvesting rate *H* (Supplementary Fig. 14), consistent with empirical findings that the largest species are the most sensitive to fishing pressure^27^,^28^. This might have been expected to result in greater production than the null case due to greater release of prey from predation, as in the case where fish species were sequentially deleted in order of decreasing body mass (Fig. 4a). However, with multispecies fishing, the prey species are fished simultaneously, thus suppressing their response to a decrease in predation. In addition, we found that multispecies sustainable yield peaked when around 60% of fish species have been lost (Fig. 5). This is higher than the percentages of collapsed species (with<10% of their unfished biomass) predicted to correspond to near-maximal multispecies yields by analyses of a suite of marine ecosystem models parameterized for 31 ecosystems, which included examination of the unselective fishing scenario^48^ (~30–40%). The peak in yield at a lower percentage predicted by these models could be because they are not fully species-resolved, unlike the model we used. This could have resulted in an underestimate of the positive effect of prey-release on functioning and yield, such that yield peaks when fewer species have collapsed.

We caution that our study has focused only on production and the abstraction of trophic interactions from communities, resulting in narrowing of the functional scope. For example, standing stock biomass, a commonly used measure of ecosystem functioning, could be considered in addition to biomass production. Although average biomass density follows largely the same trends as average production in our model, it decreases more quickly with deletions by decreasing body mass than for random deletions, in contrast to average production (Supplementary Fig. 15). The underlying reason is that species with large body masses have the slowest growth rates but tend to have large biomasses when unexploited (Supplementary Methods, Supplementary Fig. 2), so their preferential removal leads to declines in biomass that are greater than declines in production. Thus, simultaneous maintenance of biomass and production under targeted deletions of large species requires conservation of more species than if production was considered in isolation. Consideration of more types of functioning would increase the required number of species further, as would inclusion of different timescales, more locations and other types of environmental change^56^.

The results presented help to inform policy-makers on situations where arguments for biodiversity conservation based on BEF relations for provisioning ecosystem services^57^,^58^ may be weakened. Our analyses suggest that such situations are likely to be common for complex food webs. Thus, other arguments for biodiversity conservation should be considered more prominently^59^,^60^,^61^. These include conservation of biodiversity to promote the stability of ecosystems and hence the steady flow of ecosystem services^59^. In addition, there is an argument for conserving biodiversity for its own sake^60^, which is a fundamentally non-utilitarian viewpoint that might be viewed as distinct from the argument that biodiversity should be conserved because of the aesthetic enjoyment that it provides to humans.

## Methods

### Generation and validation of model food webs

The PDMM^34^, used here to predict BEF relations, simulates population dynamics in complex food webs linking thousands of species. Each model species is characterized in terms of its maturation body mass, its trophic niche as a consumer and as a resource, and its time-dependent population biomass. Consumer functional responses are of Holling type II (saturating), modified to describe prey-switching. Intra- and inter-specific competition among producers is described by Lotka–Volterra dynamics (see Supplementary Methods for a full model description). Access to the C++ code by which the PDMM is implemented can be arranged through A.G.R. on request.

The 20 model food webs were generated using 20 independent runs of the PDMM assembly algorithm, with values of 10 essential ecological properties compared with those of real large marine shelf communities (Table 1, Supplementary Methods, Supplementary Tables 2–6). We verified that all our statistical results were robust with the sample size of 20—essentially the same results were obtained with only 10 model webs. Simulated BEF relations were obtained by sequentially and randomly deleting fish species from each of the model food webs, with simulation of the population dynamics of the resulting webs until dynamic equilibria were reached. We quantified biomass production for each species population as the product of biomass intake (or consumption) rate and assimilation efficiency, after a food web had reached a population-dynamical equilibrium. In reality, ongoing environmental fluctuations will prevent ecological communities from ever reaching such equilibria. However, the equilibrium condition is used here for easy comparison with both theory^8^,^9^,^11^,^12^ and experiments^1^,^3^,^5^. The sum of biomass production of all fish species was used as a measure of ecosystem functioning, whereas the proportion of fish species remaining in a community was used to measure biodiversity.

### BEF relations under random deletions

Ten random deletion sequences were evaluated for each of the 20 model food webs, resulting in a total of 200 simulated BEF relations. Mean total fish production *P* as a function of proportion of species remaining *Φ* was computed by first averaging results for each of 300 equally spaced intervals in *Φ* between 0 and 1. This number of intervals was chosen because it ensures that each food web contributes at most one production value (averaged over 10 random deletion sequences) to each interval, so that each of the 300 averages is taken over production values that are independent. The BEF curve was then smoothed using a second degree polynomial LOESS smoother, with the span parameter chosen to minimize the corrected Akaike information criterion^62^. In addition, the smoothed relationship was fitted by two rival functions—a saturating Michaelis–Menten (MM) function and a non-saturating power-law function—using the Gauss–Newton non-linear least squares algorithm, as implemented in R^63^. Both functions have two parameters. Explicitly, the MM function is given by


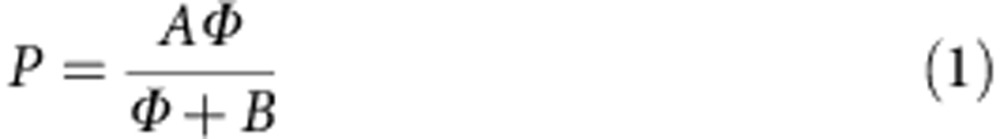


and the power-law function by


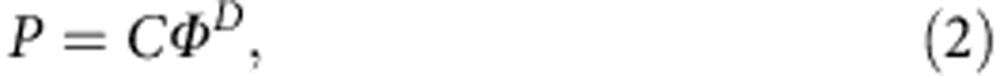


where *A*, *B*, *C* and *D* are the fitted parameters. Goodness-of-fit was assessed using the *R*^2^ statistic as well as the root mean square error.

The mean change in *P* resulting from random deletion of a species is the net effect of the direct loss of production by that species and the indirect responses in production from the remaining (undeleted) species. To understand the mechanisms underlying the shape of the BEF relation found, we first quantified the mean direct and indirect effects of the deletion of a single species in our simulations. To obtain a deeper understanding, we further partitioned the indirect effects into contributions from four categories of species, defined by their trophic relationship with the deleted species: species that were prey of the deleted species but not predators; predators but not prey; neither predators nor prey; and both predators and prey. Species were considered to be in a predator–prey relationship if the prey contributed >1% to the biomass of the predator’s diet^64^.

When drawing the relationships described, the mid-point of each *Φ* interval is plotted on the *x*-axis.

### BEF relations under ordered deletions

The random deletion scenario controls for the effects of species composition, but assumes species have equal extinction probabilities. However, fish species could have different extinction probabilities based on traits that affect vulnerability to extinction. Notably, there is evidence of preferential targeting of fish species with larger body masses^27^,^28^, higher trophic levels^29^,^30^ and larger biomasses^31^,^32^. To examine how these different trait-based extinction scenarios affect the shape of the BEF relation, we repeated the experiments but with fish species deleted according to decreasing maturation body mass, trophic level and biomass, and used the results in each case to derive a corresponding relationship between fish species richness and total fish production. The trophic level of a fish species is calculated as 1 plus the weighted mean of the trophic levels of all its prey species, with the weights being the proportional contribution of each prey species to its total consumption of biomass per year. Previous modelling studies have suggested that targeted removal of the most connected species in an ecological network results in large effects on food-web structure^33^. Therefore, to test this hypothesis, we also performed experiments where species were deleted in order of decreasing connectivity, defined as the number of species consumed by plus the number of species that consume a species.

### BEF relations under unselective multispecies harvesting

In the preceding scenarios, fish species are deleted sequentially, to quantify BEF relations across the spectrum of possible biodiversity levels. Such deletions could represent sequential targeting of species by fisheries, with the biomass of each species driven to zero or close to zero before another species is targeted. However, fisheries often cause mortality of multiple species at once, for example by using trawls or purse seines^65^. Therefore, we also performed experiments on each of the 20 food webs where all fish species experience a constant harvesting rate *H*, defined as the rate of removal of the total biomass of a fish species population by fishing. *H* was varied in the experiments from 0.06 year^−1^ to 8 year^−1^ in increments of 0.02 year^−1^. The lower limit reflects the lowest value of *H* found from fishing regimes in the Celtic and North Seas^35^, whereas the upper limit ensures removal of all fish species in all webs. In each experiment, fishing was applied to all fish species in a model food web until a new community equilibrium was reached, at which point the fish species richness and total fish production were both recorded. All species with a maturation body mass above 10^−3^ kg were considered fish species (see Supplementary Methods for a detailed justification and discussion). The recorded richness and production values from all experiments were then used to derive the average BEF relation, as in the sequential deletion scenarios. In addition, the sustainable yield in each experiment was calculated as the equilibrium total fish biomass multiplied by *H*. These yields were then used together with the richness values to produce a relationship between richness and mean yield.

## Author contributions

T.F., A.G.R. and K.D.F. conceived and designed the research; T.F. and A.G.R. performed the simulations; A.G.R. provided model code; and T.F. and A.G.R. analysed data from the simulations. All authors made substantial contributions to discussing the results and writing the manuscript.

## Additional information

**How to cite this article:** Fung, T. *et al*. Impact of biodiversity loss on production in complex marine food webs mitigated by prey-release. *Nat. Commun.* 6:6657 doi: 10.1038/ncomms7657 (2015).

## Supplementary Material

Supplementary Information Supplementary Figures 1-15, Supplementary Tables 1-6, Supplementary Methods and Supplementary References

## Figures and Tables

**Table 1:** Validation of model food webs.

**Property**	**Range of model values**	**Range of empirically derived values**
Phytoplankton species richness	2,559–2,961	268–1,700
Fish species richness	148–280	192–314
Dietary diversity of fish species	6.17–8.05	6–14
Diet-partitioning exponent for fish species^36^	0.509–0.644	0.21–0.66
Maturation body mass of phytoplankton species (kg)	10^−14.7^–10^−9.01^	10^−15^–10^−8.69^
Maturation body mass of fish species (kg)	10^−3.0^–10^2.47^	10^−3.0^–10^2.54^
Trophic level of fish species	2.03–5.53	2–4.53
Slope of diversity spectrum	0.149–0.491	0.163–0.460
Slope of biomass size-spectrum	−0.536–0.0234	−0.25–0.025
Biomass density of fish species (kg m^−2^)	10^−13.0^–10^−1.60^	10^−10.1^–10^−2.28^

Range of values of 10 key properties for the 20 PDMM food webs used, compared with empirically derived ranges pertaining to temperate shelf communities. In calculating the slopes of the diversity spectra, a lower bound of 1 kg was used for 16 of the 20 food webs, whereas a lower bound of 3–35 kg was used for the remaining four webs.
