# Supplementary material for: Impact of biodiversity loss on production in complex marine food webs mitigated by prey-release
Source: Nat Commun. 2015 Mar 23;6:6657. doi: 10.1038/ncomms7657 (PMC4382996; doi:10.1038/ncomms7657)
Supplement: Supplementary Information — Supplementary Figures 1-15, Supplementary Tables 1-6, Supplementary Methods and Supplementary References [file ncomms7657-s1.pdf]

Supplementary Figures

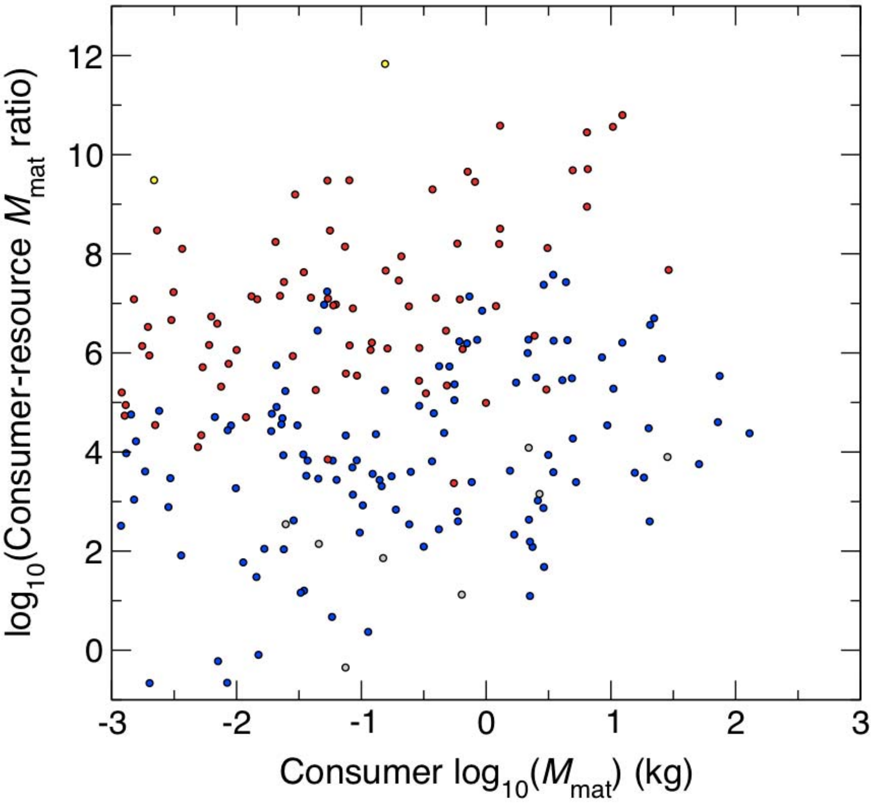

Supplementary Figure 1. **Realised consumer-resource maturation body mass ratios for fish species in a PDMM food web.** Realised consumer-resource maturation body mass (M_{mat}) ratio against M_{mat} for all 206 fish species in one of the 20 PDMM temperate shelf food webs used (corresponding to Model food web 1 in Supplementary Table 3). Each data point is colored according to the trophic level of the species it represents, rounded to the nearest integer. Rounded trophic levels of 2, 3, 4 and 5 are represented by yellow, red, blue and grey respectively.

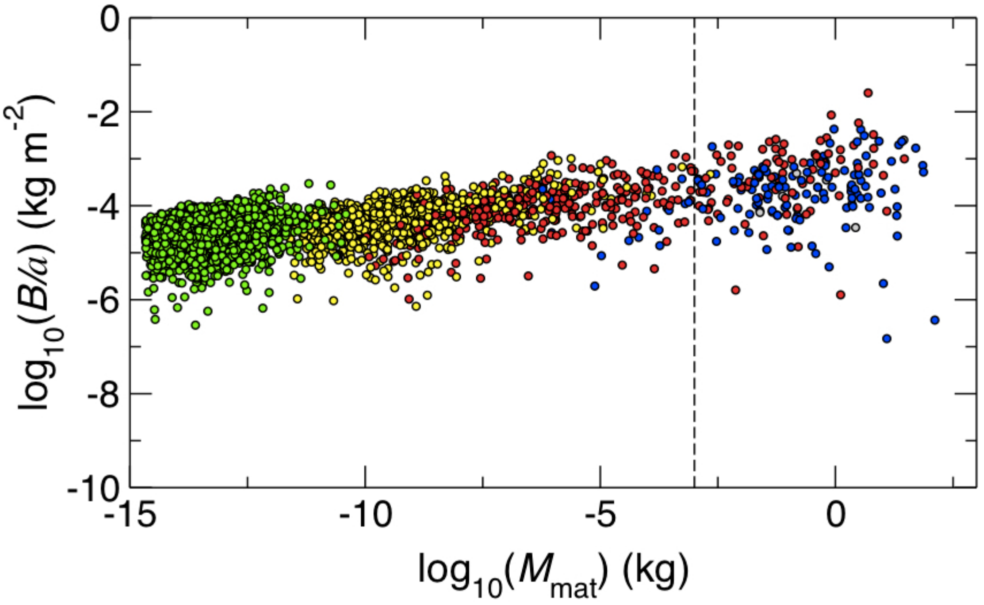

Supplementary Figure 2. **Body mass-biomass distribution for species in a PDMM food web.** Biomass density (biomass, *B*, divided by system area, *a*) against maturation body mass (M_{mat}) for all species in one of the 20 PDMM temperate shelf food webs used (corresponding to Model food web 1 in Supplementary Table 3). Each data point is colored according to the trophic level of the species it represents, rounded to the nearest integer. Rounded trophic levels of 1, 2, 3, 4 and 5 are represented by green, yellow, red, blue and grey respectively. The dashed vertical line denotes the minimum M_{mat} threshold above which a model species is categorised as a fish species.

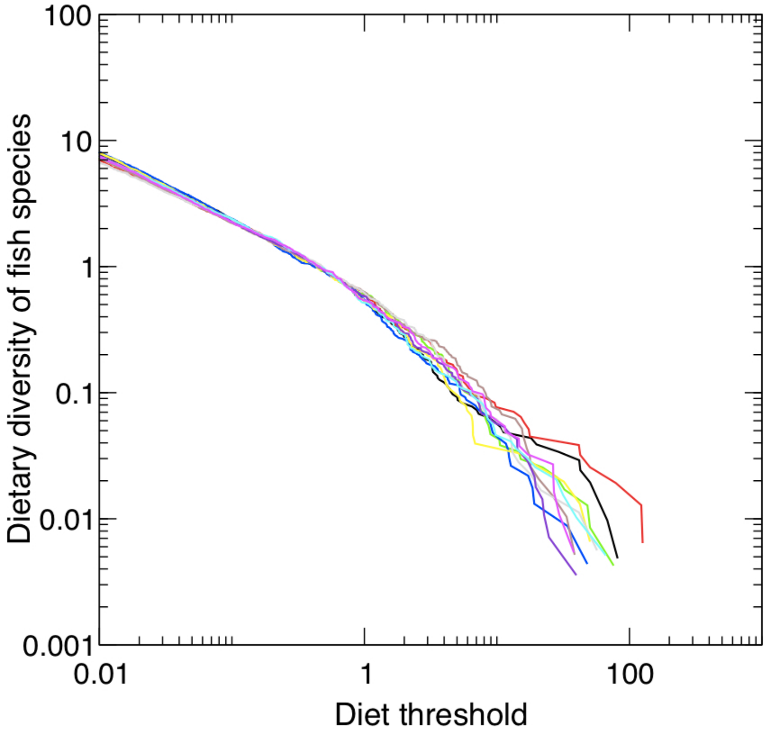

Supplementary Figure 3. **Diet-partitioning functions for 10 PDMM food webs**. Dietary diversity of fish species against the diet threshold for 10 of the 20 PDMM temperate shelf food webs used (corresponding to Model food webs 1-10 in Supplementary Tables 3 and 4). The dietary diversity is measured as the average number of resource species consumed by a fish species, where a species is counted as a resource if the logit of its proportional diet contribution is above the logarithm of the diet threshold.

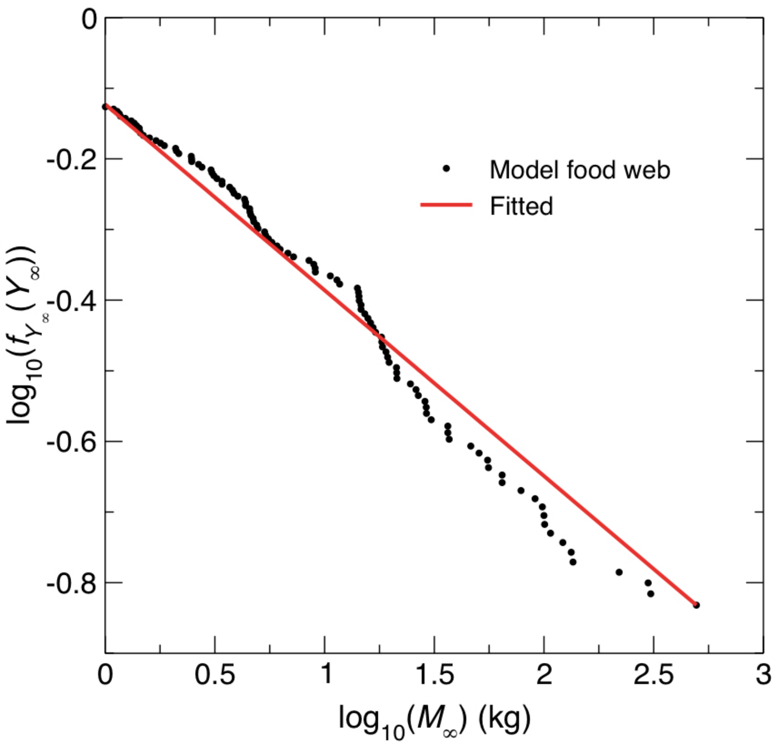

Supplementary Figure 4. **Diversity spectrum for a PDMM food web**. Probability density function of asymptotic body masses on a $\log_{10}$ scale ($f_{Y_\infty}(Y_\infty)$, where $Y_\infty = \log_{10}(M_\infty)$ and M_∞ is the asymptotic body mass) against M_∞ for all 206 fish species in one PDMM temperate shelf food web used (corresponding to Model food web 1 in Supplementary Table 3). The function derived from a fitted truncated Pareto distribution is also shown.

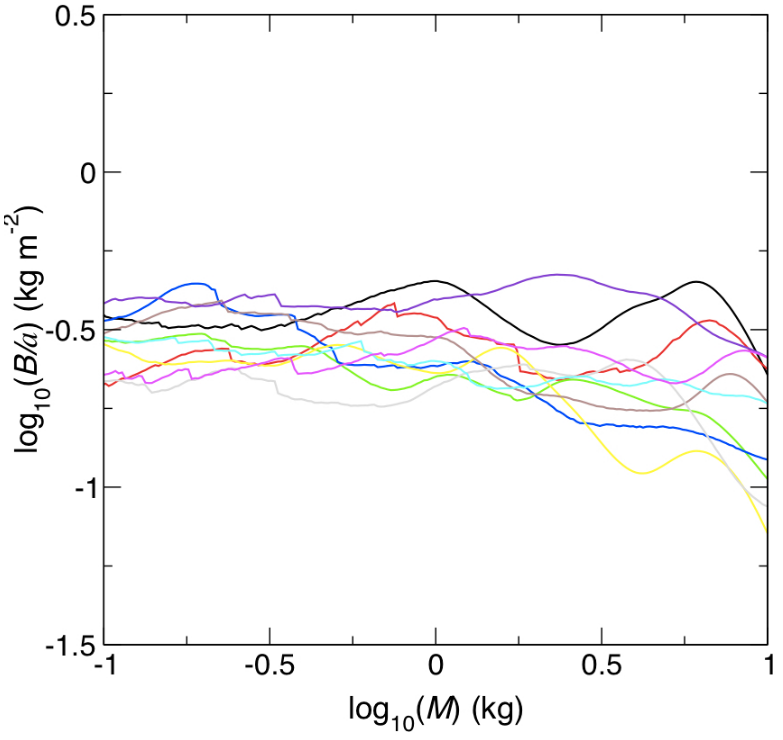

Supplementary Figure 5. **Biomass size-spectra for 10 PDMM food webs**. Biomass density (biomass, *B*, divided by system area, *a*) against individual body mass (*M*) for fish species in 10 of the 20 PDMM temperate shelf food webs used (corresponding to Model food webs 1-10 in Supplementary Tables 3 and 4).

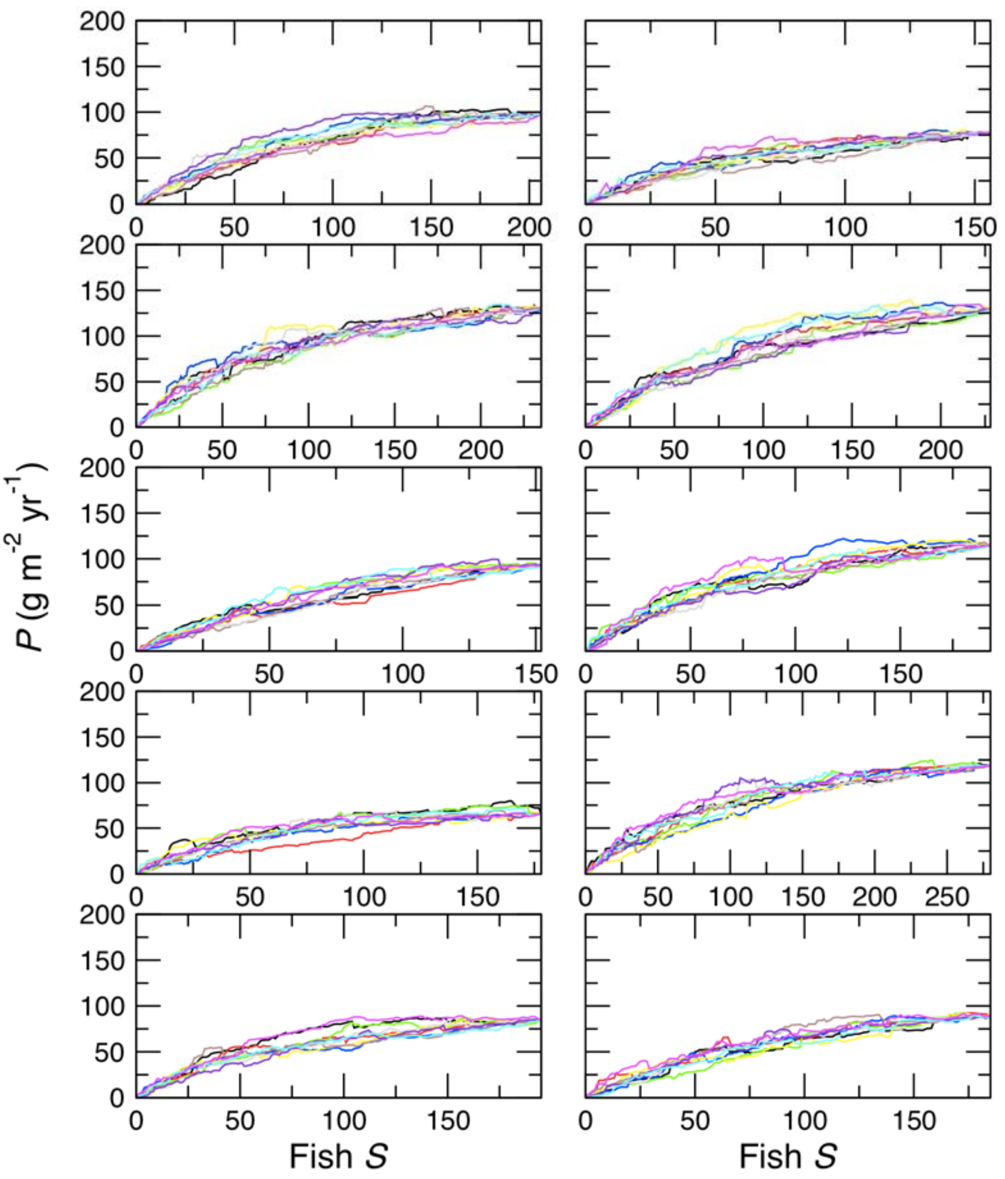

Supplementary Figure 6. **Richness-production relations produced from all deletion sequences for the first 10 PDMM food webs**. Total fish biomass production P against fish species richness S for each of the 100 simulated random deletion sequences performed for the first 10 PDMM temperate shelf food webs used (corresponding to Model food webs 1-10 in Supplementary Tables 3 and 4; 10 deletion sequences were simulated for each web).

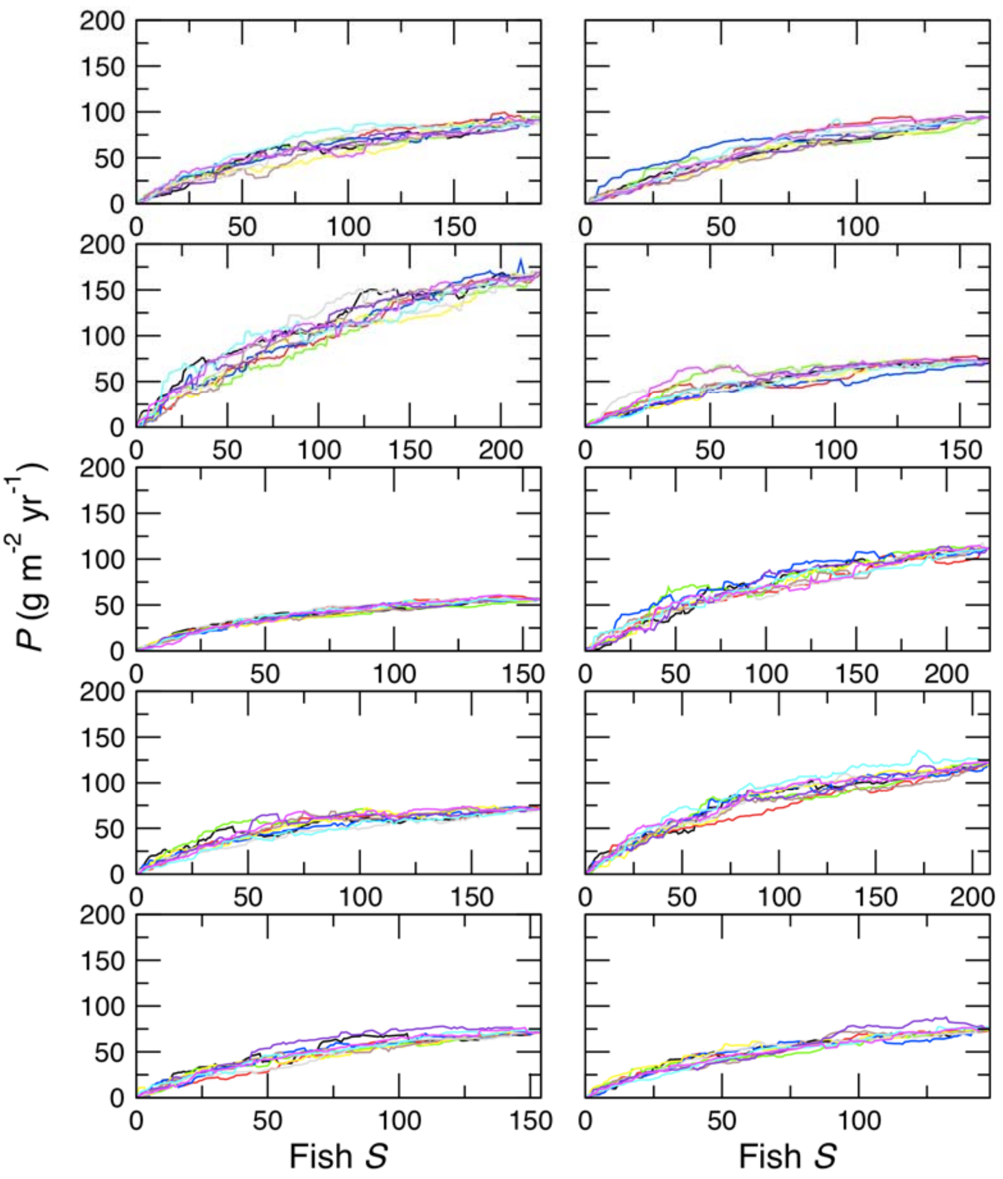

Supplementary Figure 7. **Richness-production relations produced from all deletion sequences for the last 10 PDMM food webs**. Total fish biomass production P against fish species richness S for each of the 100 simulated random deletion sequences performed for the last 10 PDMM temperate shelf food webs used (corresponding to Model food webs 11-20 in Supplementary Tables 5 and 6; 10 deletion sequences were simulated for each web).

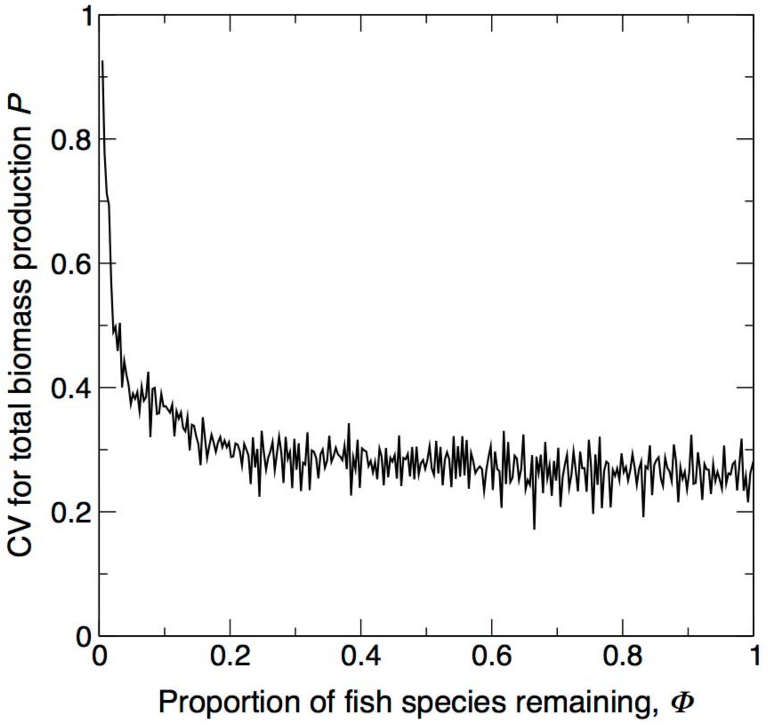

Supplementary Figure 8. Coefficient of variation for total fish biomass production against normalised fish species richness for random deletions. The coefficient of variation (CV) in total fish biomass production *P* at each normalised richness value was derived from the 200 random deletion sequences for all 20 PDMM temperate shelf food webs.

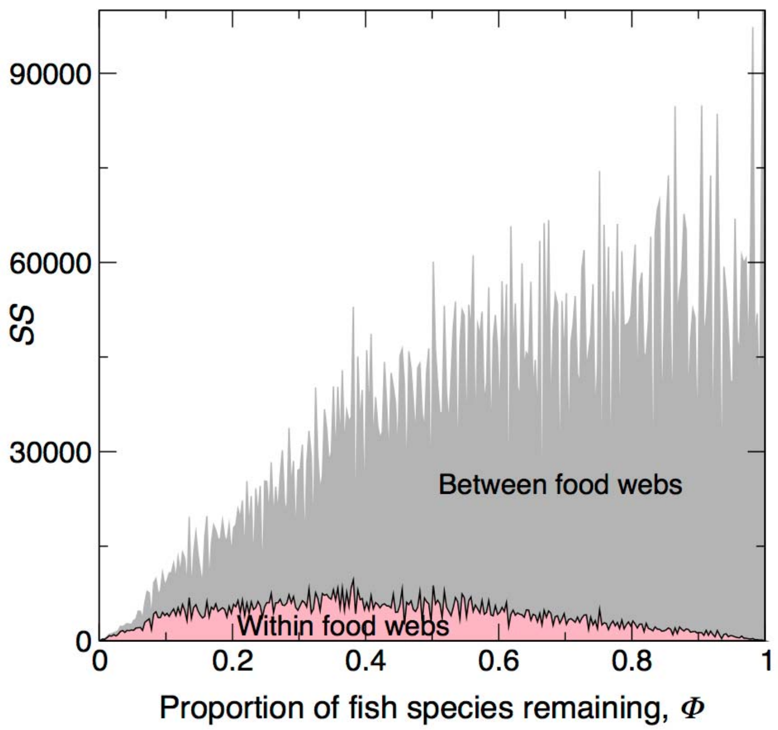

Supplementary Figure 9. Partitioning of variance in total fish biomass production across normalised fish species richness for random deletions. Sum of squared differences between total fish biomass production values and the mean (*SS*) across all normalised fish species richness values, derived from the 200 random deletion sequences for all 20 PDMM temperate shelf food webs. *SS* was partitioned into variation in production between and within food webs.

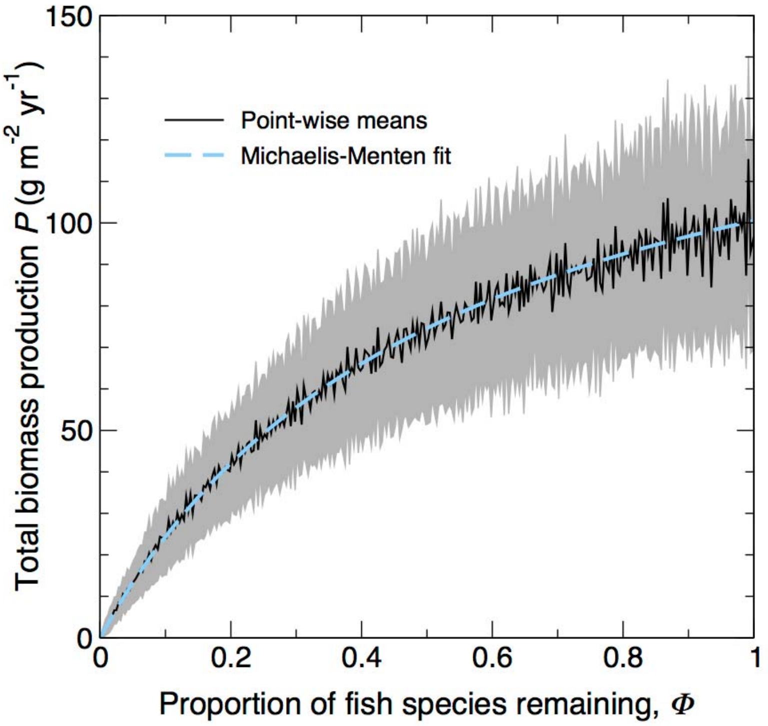

Supplementary Figure 10. Total fish biomass production against normalised fish species richness for random deletions. Point-wise means (black), standard deviations (grey) and a fitted Michaelis-Menten function (light blue), based on the 200 random deletion sequences for all 20 PDMM temperate shelf food webs (10 sequences per web).

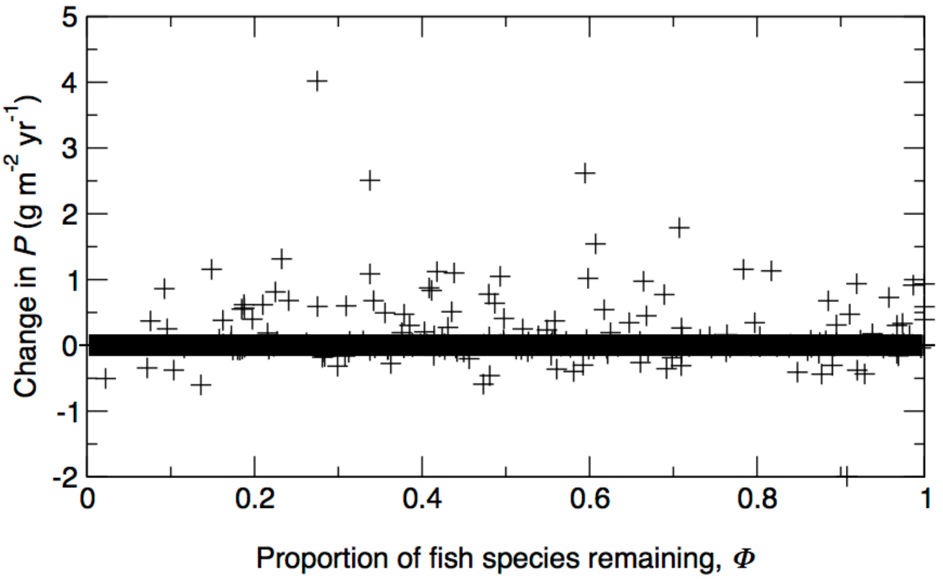

Supplementary Figure 11. Change in production due to species that were both prey and predators of deleted species. Contributions to the change in total fish biomass production P from species that were both prey and predators of deleted species, derived from the 200 random deletion sequences for all 20 PDMM temperate shelf food webs (10 sequences per web). These species only occurred in 214 out of a total of 30,100 deletions; thus, contributions were zero in the vast majority of cases (the cluster of crosses at zero forms the thick, black horizontal line). The mean absolute contribution, 0.00216 g m^{-2} yr^{-1}, is 125 times smaller than the mean absolute contribution from all other undeleted species (the "Prey of deleted", "Predators of deleted" and "Neither" species in Fig. 2b of the main text). Therefore, contributions from species that were both prey and predators of deleted species were negligible.

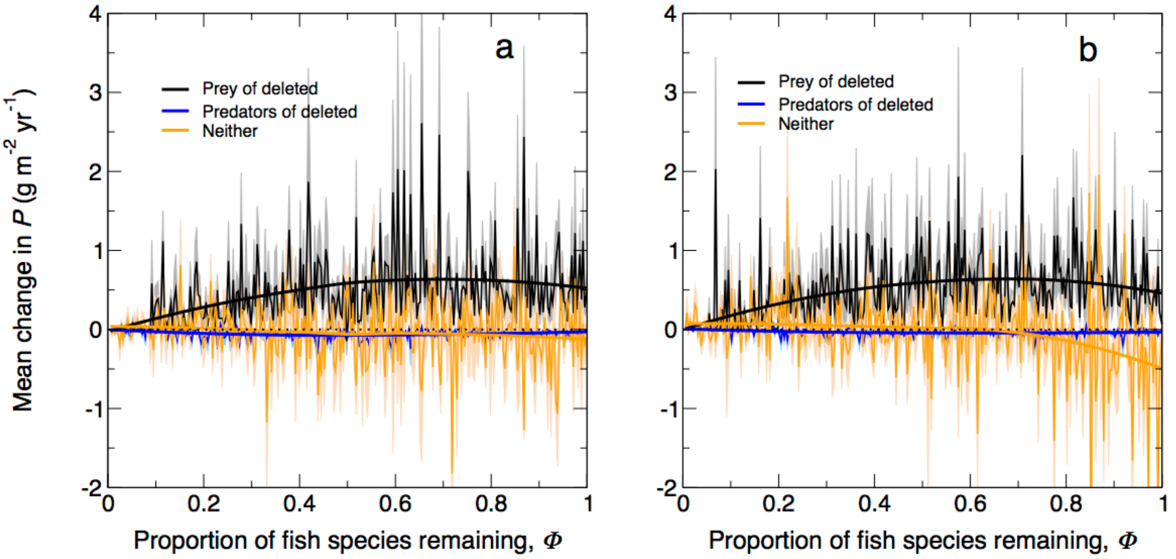

Supplementary Figure 12. Components of total change in fish biomass production following ordered species deletions. The change in production (P) from the responses of undeleted species to species deletions are split into those from prey and predators of the deleted species as well as from species that were neither prey nor predators, for **a**, deletions by decreasing body mass and **b**, decreasing trophic level. Each panel shows point-wise means (thin lines) with standard errors (pale colours) and LOESS smoothers (thick lines), based on 20 ordered deletions for all 20 PDMM temperate shelf food webs. Note the wider y-axis scale compared with that in Fig. 2b.

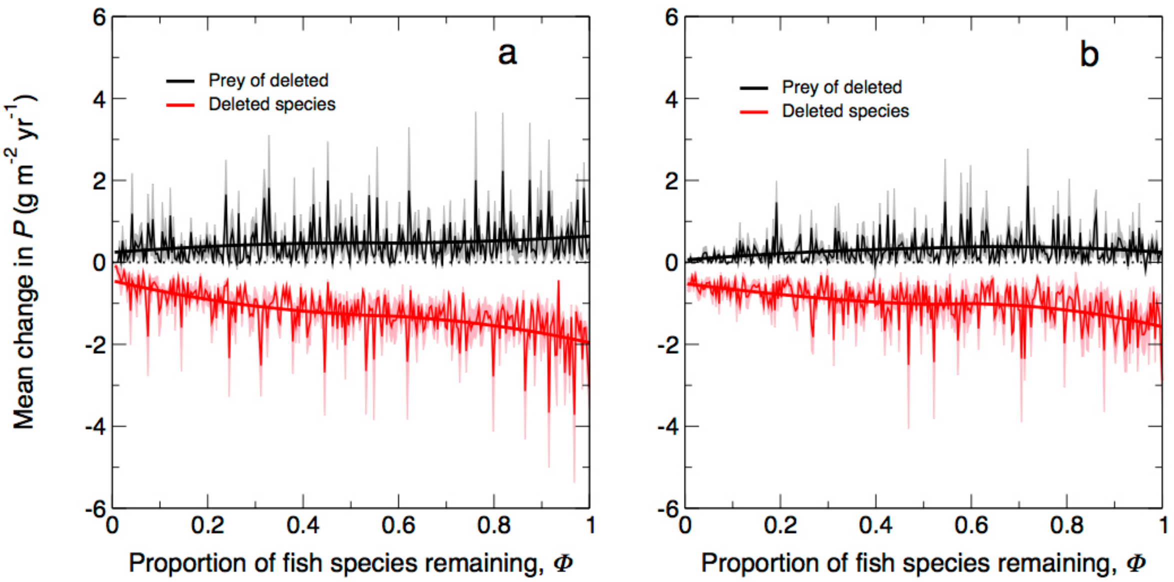

Supplementary Figure 13. Change in production due to species that were prey of the deleted species and due to the deleted species. The change in total fish biomass production (*P*) from the responses of undeleted species that were prey of the deleted species as well as from loss of the deleted species, for **a**, deletions by decreasing biomass and **b**, decreasing connectivity. Each panel shows point-wise means (thin lines) with standard errors (pale colours) and LOESS smoothers (thick lines), based on 20 ordered deletions for all 20 PDMM temperate shelf food webs.

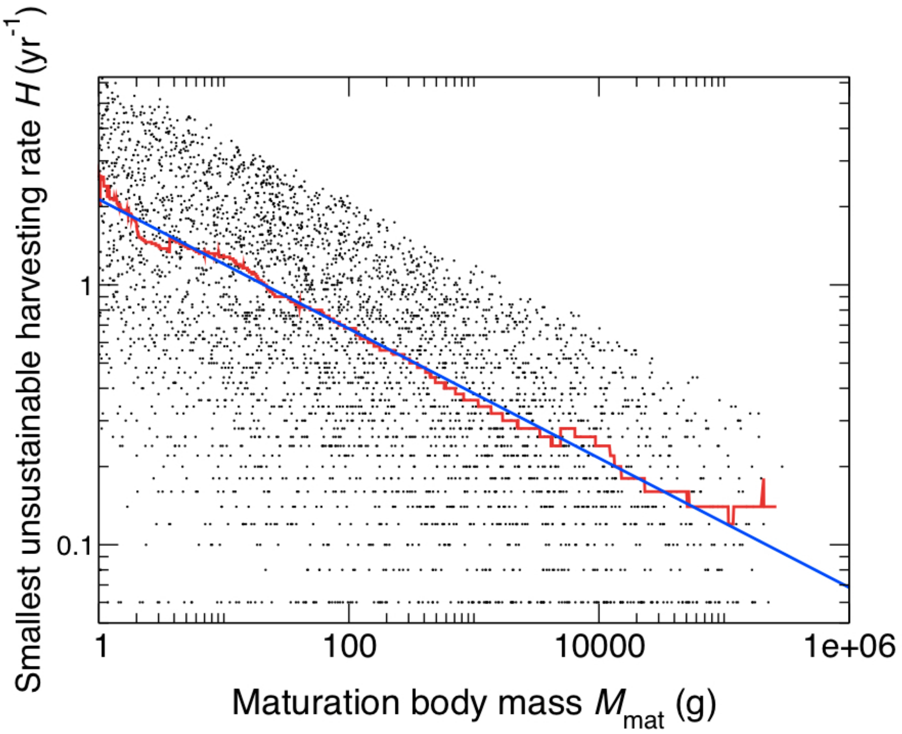

Supplementary Figure 14. Smallest unsustainable harvesting rate against maturation body mass under unselective multispecies fishing. In the multispecies fishing scenario, the smallest harvesting rates (H) causing the extinction of each fish species in the 20 PDMM food webs are plotted against their maturation body masses. The red line is a 1,001 point running median whereas the blue line is a two-parameter median regression line given by $H = 2.13\left(M_{\text{mat}}\right)^{-0.249}$. Spearman's rank correlation coefficient between the two variables ranges from -0.75 to -0.44 for the 20 food webs.

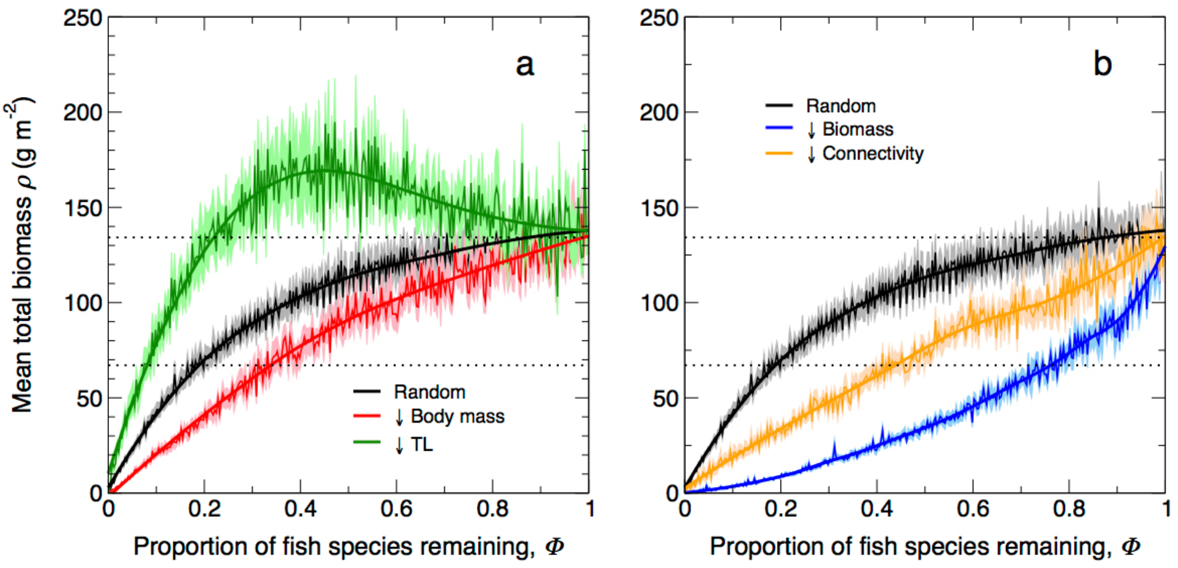

Supplementary Figure 15. Predicted mean total fish biomass against normalised fish species richness for random and ordered deletions. Biomass-richness relations are shown for **a**, random deletions and deletions by decreasing body mass and trophic level, and **b**, random deletions and deletions by decreasing biomass and connectivity. For each relation, point-wise means (thin lines), standard errors (pale colours) and LOESS smoothers (thick lines) are presented, based on the 20 ordered deletions for all 20 food webs. The two dotted horizontal lines mark the initial total fish biomass and 50% of this value.

Supplementary Tables

Parameter	Definition	Value	Data Source(s)
Parameters determining trait values of new species			
d	Determines rate of evolution of M_{mat}	2.5	
D	Niche space dimension	5	
M_{min}	Minimum M_{mat} of a model species	10^{-15} kg	Beardall et al.[1]; Fenchel & Finlay[2]
M_{max}	Maximum M_{mat} of a model species	$10^{2.54}$ kg	Froese & Pauly[3]
p	Determines number of new species per iteration of PDMM assembly algorithm	0.01	
r_F, r_V	Radii of hyperspheres for the foraging traits (r_F) and vulnerability traits (r_V)	100, 5.08	

μ_F, μ_V	Control phylogenetic correlations of foraging traits (μ_F) and vulnerability traits (μ_V)	1.5, 1.4	
Parameters specifying food-web dynamics			
a	Area occupied by modelled food web	6×10^{11} m^2	Sea Around Us Project[4]
C_b	Prefactor for consumer attack rate	1.86×10^{-7} kg$^{-3/4}$ yr^{-1}	
C_{lc}	Prefactor for consumer metabolic loss rate	0.259 kg$^{1/4}$ yr^{-1}	Brown et al.[5]
$C_{r\max}$	Prefactor for consumer maximum net growth rate	$5C_{lc}$	
$C_{\sigma\max}$	Prefactor for producer maximum net growth rate	0.182 kg$^{1/4}$ yr^{-1}	Niklas & Enquist[6]
$P_{N,\max}$	Maximum NPP of a single producer species in monoculture per unit planar area	0.055 kg m^{-2} yr^{-1}	Sea Around Us Project[4]; Peters[7]
p_κ	Probability of one producer species having a negative antagonistic effect on another	0.0025	
w_c	Consumer niche width	0.75	

w_s	Switching similarity width	0.75	
α_c	Determines how quickly trophic interaction strength decreases when consumer-resource M_{mat} ratio increases above preferred ratio, η_c	0.07	
β_c	Determines how quickly trophic interaction strength decreases when consumer-resource M_{mat} ratio decreases below preferred ratio, η_c	0.25	
η_c	Preferred consumer-resource M_{mat} ratio	10^2	Jennings et al.[8,9]
ε	Consumer assimilation efficiency	0.6	Hendriks[10]
κ	Strength of negative antagonistic effect of one producer species on another	0.4	
ζ_b	Allometric exponent for consumer attack rate	-0.25	Peters[7]; Ware[11]; Andersen & Beyer[12]

ζ_{lc}	Allometric exponent for consumer metabolic loss rate	-0.25	Brown et al.[5]
$\zeta_{r\max}$	Allometric exponent for consumer maximum net growth rate	-0.25	Savage et al.[13]
$\zeta_{\sigma\max}$	Allometric exponent for producer maximum net growth rate	-0.25	Niklas & Enquist[6]

Supplementary Table 1. List of PDMM parameters with definitions and values, together with data sources used to derive values. M_{mat} is the maturation body mass of a species.

Property	Data source(s) for empirically derived values	Study locations pertaining to empirically derived values
Phytoplankton S	Smith et al.[14]; Ojaveer et al.[15]	142 natural and 239 experimental aquatic ecosystems, Baltic Sea
Fish S	Froese & Pauly[3]	Celtic-Biscay Shelf, North Sea
Dietary diversity of fish spp.	Rossberg et al.[16]	North Sea, Eastern Bering Sea,
Diet-partitioning exponent for fish spp.	Rossberg et al.[16]; Rossberg[17]	Northwest Atlantic Shelf, Open North Atlantic, Open Tropical Atlantic, South China Sea
M_{mat} of phytoplankton spp.	Beardall et al.[1]; Barnes et al.[18]	North Sea, Irminger Sea, Benguela upwelling, Norwegian Sea, 5 unspecified locations in Atlantic Ocean
M_{mat} of fish spp.	Froese & Pauly[3]	Celtic-Biscay Shelf, North Sea
Trophic level of fish spp.	Froese & Pauly[3]	Celtic-Biscay Shelf, North Sea
Slope of diversity spectrum	Reuman et al.[19]	Celtic-Biscay Shelf, North Sea
Slope of biomass size-spectrum	Jennings & Blanchard[20]	North Sea
B/a of fish species	Fung et al.[21]	North Sea

Supplementary Table 2. **Data sources and study locations for empirically derived values of 10 key food-web properties.** S denotes "species richness", M_{mat} denotes "Maturation body mass" and B/a denotes "Biomass density" (Biomass, B, divided by system area, a).

Property	Model food web 1	Model food web 2	Model food web 3	Model food web 4	Model food web 5	Empirical range
Phytoplankton S	2,708	2,841	2,700	2,715	2,726	268 - 1,700
Fish S	206	156	235	228	152	192 - 314
Dietary diversity of fish spp.	7.59	7.36	7.25	8.05	7.92	6 - 14
Diet-partitioning exponent for fish spp.	0.624	0.581	0.605	0.644	0.631	0.21 - 0.66
M_{mat} of phytoplankton spp. (kg)	$10^{-14.7}$ - $10^{-10.4}$	$10^{-14.7}$ - $10^{-10.9}$	$10^{-14.7}$ - $10^{-9.01}$	$10^{-14.7}$ - $10^{-10.9}$	$10^{-14.7}$ - $10^{-10.6}$	10^{-15} - $10^{-8.69}$
M_{mat} of fish spp. (kg)	$10^{-2.93}$ - $10^{2.11}$	$10^{-2.99}$ - $10^{2.32}$	$10^{-2.97}$ - $10^{2.23}$	$10^{-2.99}$ - $10^{1.71}$	$10^{-2.99}$ - $10^{1.68}$	10^{-3} - $10^{2.54}$
Trophic level of fish spp.	2.08 - 4.99	2.15 - 5.32	2.03 - 5.44	2.48 - 5.22	2.19 - 4.94	2 - 4.35
Slope of diversity spectrum	0.263	0.214	0.406	0.287	0.472	0.163 - 0.460
Slope of biomass size-spectrum	0.0145	0.0178	–0.161	–0.274	–0.220	–0.25 - 0.025
B/a of fish species (kg m^{-2})	$10^{-6.83}$ - $10^{-1.60}$	$10^{-7.55}$ - $10^{-1.89}$	$10^{-11.9}$ - $10^{-2.32}$	$10^{-6.95}$ - $10^{-2.00}$	$10^{-10.3}$ - $10^{-2.25}$	$10^{-10.1}$ - $10^{-2.28}$

Supplementary Table 3. **Values of 10 key properties for five of the 10 PDMM food webs used, compared with empirically derived values**. The five model food webs were produced from the first five of 20 runs of the PDMM assembly algorithm, whereas the empirically derived values pertain to temperate shelf communities in the Northeast Atlantic. S denotes "species richness", M_{mat} denotes "Maturation body mass" and B/a denotes "Biomass density" (Biomass, B, divided by system area, a). In calculating the slopes of the diversity spectra, lower bounds of 1 kg were used except for Model food web 2, for which a lower bound of 5 kg was used.

Property	Model food web 6	Model food web 7	Model food web 8	Model food web 9	Model food web 10	Empirical range
Phytoplankton S	2,559	2,785	2,758	2,850	2,859	268 - 1,700
Fish S	193	178	280	195	185	192 - 314
Dietary diversity of fish spp.	7.22	6.67	7.49	7.33	7.36	6 - 14
Diet-partitioning exponent for fish spp.	0.592	0.581	0.617	0.617	0.606	0.21 - 0.66
M_{mat} of phytoplankton spp. (kg)	$10^{-14.7}$ - $10^{-9.86}$	$10^{-14.7}$ - $10^{-10.7}$	$10^{-14.7}$ - $10^{-11.0}$	$10^{-14.7}$ - $10^{-10.7}$	$10^{-14.7}$ - $10^{-10.4}$	10^{-15} - $10^{-8.69}$
M_{mat} of fish spp. (kg)	$10^{-2.97}$ - $10^{2.30}$	$10^{-2.99}$ - $10^{2.28}$	$10^{-2.98}$ - $10^{2.12}$	$10^{-3.00}$ - $10^{2.47}$	$10^{-3.00}$ - $10^{2.10}$	10^{-3} - $10^{2.54}$
Trophic level of fish spp.	2.35 - 4.98	2.10 - 5.09	2.21 - 5.19	2.36 - 5.25	2.06 - 5.28	2 - 4.35
Slope of diversity spectrum	0.398	0.196	0.244	0.227	0.149	0.163 - 0.460
Slope of biomass size-spectrum	–0.183	–0.0630	–0.0220	–0.0982	0.0234	–0.25 - 0.025
B/a of fish species (kg m^{-2})	$10^{-9.68}$ - $10^{-2.03}$	$10^{-9.23}$ - $10^{-2.10}$	$10^{-12.9}$ - $10^{-2.04}$	$10^{-7.75}$ - $10^{-2.19}$	$10^{-7.59}$ - $10^{-2.15}$	$10^{-10.1}$ - $10^{-2.28}$

Supplementary Table 4. **Values of 10 key properties for five of the 10 PDMM food webs used, compared with empirically derived values**. The five model food webs were produced from the second five of 20 runs of the PDMM assembly algorithm, whereas the empirically derived values pertain to temperate shelf communities in the Northeast Atlantic. S denotes "species richness", M_{mat} denotes "Maturation body mass" and B/a denotes "Biomass density" (Biomass, B, divided by system area, a). In calculating the slopes of the diversity spectra, a lower bound of 1 kg was used for all model food webs.

Property	Model food web 11	Model food web 12	Model food web 13	Model food web 14	Model food web 15	Empirical range
Phytoplankton S	2,809	2,854	2,618	2,765	2,802	268 - 1,700
Fish S	191	149	222	162	157	192 - 314
Dietary diversity of fish spp.	7.63	7.11	7.56	7.10	6.17	6 - 14
Diet-partitioning exponent for fish spp.	0.602	0.578	0.591	0.566	0.509	0.21 - 0.66
M_{mat} of phytoplankton spp. (kg)	$10^{-14.7}$ - $10^{-10.4}$	$10^{-14.7}$ - $10^{-11.2}$	$10^{-14.7}$ - $10^{-10.4}$	$10^{-14.7}$ - $10^{-10.4}$	$10^{-14.7}$ - $10^{-10.0}$	10^{-15} - $10^{-8.69}$
M_{mat} of fish spp. (kg)	$10^{-2.99}$ – $10^{1.91}$	$10^{-2.93}$ – $10^{1.78}$	$10^{-3.00}$ - $10^{2.04}$	$10^{-2.97}$ - $10^{2.15}$	$10^{-2.97}$ - $10^{2.23}$	10^{-3} - $10^{2.54}$
Trophic level of fish spp.	2.03 - 5.23	2.22 - 5.08	2.06 - 5.38	2.13 - 4.91	2.25 - 5.35	2 - 4.35
Slope of diversity spectrum	0.491	0.472	0.270	0.266	0.204	0.163 - 0.460
Slope of biomass size-spectrum	–0.351	–0.536	–0.232	–0.0189	0.0133	–0.25 - 0.025
B/a of fish species (kg m^{-2})	$10^{-10.5}$ - $10^{-2.21}$	$10^{-10.2}$ - $10^{-2.23}$	$10^{-13.0}$ - $10^{-1.68}$	$10^{-9.62}$ - $10^{-1.76}$	$10^{-9.04}$ - $10^{-1.85}$	$10^{-10.1}$ - $10^{-2.28}$

Supplementary Table 5. **Values of 10 key properties for five of the 20 PDMM food webs used, compared with empirically derived values**. The five model food webs were produced from the third five of 20 runs of the PDMM assembly algorithm, whereas the empirically derived values pertain to temperate shelf communities in the Northeast Atlantic. S denotes "species richness", M_{mat} denotes "Maturation body mass" and B/a denotes "Biomass density" (Biomass, B, divided by system area, a). In calculating the slopes of the diversity spectra, lower bounds of 1 kg were used except for Model food web 15, for which a lower bound of 3 kg was used.

Property	Model food web 16	Model food web 17	Model food web 18	Model food web 19	Model food web 20	Empirical range
Phytoplankton S	2,745	2,961	2,772	2,865	2,891	268 - 1,700
Fish S	224	181	209	154	148	192 - 314
Dietary diversity of fish spp.	6.76	7.10	7.20	7.05	6.74	6 - 14
Diet-partitioning exponent for fish spp.	0.553	0.568	0.564	0.587	0.550	0.21 - 0.66
M_{mat} of phytoplankton spp. (kg)	$10^{-14.7}$ - $10^{-11.0}$	$10^{-14.7}$ - $10^{-10.9}$	$10^{-14.7}$ - $10^{-10.7}$	$10^{-14.7}$ - $10^{-10.8}$	$10^{-14.7}$ - $10^{-10.5}$	10^{-15} - $10^{-8.69}$
M_{mat} of fish spp. (kg)	$10^{-3.00}$ - $10^{2.35}$	$10^{-2.96}$ - $10^{1.96}$	$10^{-2.99}$ - $10^{2.15}$	$10^{-2.97}$ - $10^{1.89}$	$10^{-2.96}$ - $10^{1.93}$	10^{-3} - $10^{2.54}$
Trophic level of fish spp.	2.31 - 5.29	2.30 - 4.97	2.15 - 5.41	2.40 - 4.80	2.08 - 5.53	2 - 4.35
Slope of diversity spectrum	0.207	0.257	0.397	0.490	0.350	0.163 - 0.460
Slope of biomass size-spectrum	–0.0051	–0.170	–0.199	–0.383	–0.243	–0.25 - 0.025
B/a of fish species (kg m^{-2})	$10^{-10.7}$ - $10^{-1.74}$	$10^{-8.19}$ - $10^{-2.35}$	$10^{-9.96}$ - $10^{-1.83}$	$10^{-7.75}$ - $10^{-2.19}$	$10^{-5.51}$ - $10^{-2.11}$	$10^{-10.1}$ - $10^{-2.28}$

Supplementary Table 6. **Values of 10 key properties for five of the 20 PDMM food webs used, compared with empirically derived values**. The five model food webs were produced from the last five of 20 runs of the PDMM assembly algorithm, whereas the empirically derived values pertain to temperate shelf communities in the Northeast Atlantic. S denotes "species richness", M_{mat} denotes "Maturation body mass" and B/a denotes "Biomass density" (Biomass, B, divided by system area, a). In calculating the slopes of the diversity spectra, lower bounds of 1 kg were used except for Model food webs 16 and 20, for which lower bounds of 35 kg and 4 kg were used, respectively.

Supplementary Methods

Further details on the Population-Dynamical Matching Model (PDMM)

Overview of PDMM: Model elements. The PDMM was originally designed to extend the understanding of food-web topology represented by the Matching Model[22], by adding an explicit representation of interspecific size-structure and biomass dynamics through a system of differential equations[23]. Food-web topology and the strengths of trophic (feeding) interactions in the PDMM are determined by the body sizes of consumer and resource species together with abstract trophic traits of consumers and resources[24]. These emerge from a community assembly process consisting of a cycle of species introductions and simulation of food-web dynamics to a new equilibrium[23]. In subsequent studies, the PDMM was developed and used to represent temperate shelf communities in the Northeast Atlantic[25–27]. The model has been shown to reproduce structural[26] and dynamic[25,27] properties of temperate shelf food webs. For the present study we have incorporated additional minor modifications to the PDMM to improve control over species richness in mature communities during community assembly and to increase its performance in reaching equilibrium dynamics. In every other aspect, it is identical to the PDMM implementation in Fung et al.[26] In the remainder of this subsection and the next two subsections ("Overview of PDMM: Community assembly" and "Overview of PDMM: Population dynamics and food-web structure"), the structure of the PDMM is summarised. The next three subsections ("Detailed description of PDMM: Community assembly", "Detailed Description of PDMM: Population dynamics of producer species" and "Detailed Description of PDMM: Population dynamics of consumer species") give a complete model description, highlighting changes

made in comparison with Fung et al.[26] The parameter values used for the PDMM implementation in the present study are also largely the same as those in Fung et al.[26], except for changes arising from the minor modifications made. These changes in parameterisation of the PDMM are described in the last subsection of this section ("Model parameterisation").

Overview of PDMM: Community assembly. The PDMM uses a stochastic, iterative assembly algorithm to construct dynamic model food webs with many species and realistic interspecific body size and trophic link structures[23,26]. One model food web is produced from each run of the algorithm. The algorithm starts with an empty model food web (with no species) and then repeatedly adds sets of new species to this web, representing invasions[23,26]. Apart from the first set of species, which have body sizes that take fixed default values and abstract trophic traits with values chosen from random distributions, new species have trait values that are determined by randomly changing those of a species already found in the food web. This has the effect of implicitly emulating the effect of phylogenetic structure in a hypothetical pool of species from which species invade, as well as evolution of species in this pool[28]. The resulting phylogenetic correlations of the trait values of species within the food web have been found to be important determinants of food-web structure[22,29–31]. In between each set of species additions, food-web biomass dynamics are simulated to represent the dynamic response of the food web to the species additions[23,26]. During simulation of dynamics, extinct species are removed from the food web, representing a natural selection filter for the species subset that can coexist dynamically. The resulting food web emerges from a simulation of natural dynamic evolutionary and population processes, rather than being pre-specified. This gradual assembly of model food webs using a developmental approach (*sensu* Taylor[32]) enables the construction of food webs with up to thousands of dynamically coexisting species[26].

Overview of PDMM: Population dynamics and food-web structure. Food-web dynamics in the PDMM are specified by linked differential equations that describe gains and losses in the biomass of each species' population due to trophic interactions, metabolism and natural mortality. Equations for producers differ from those for consumers, to reflect their differences in energy acquisition and growth. Specifically, if the *S* species in a PDMM food web are indexed such that the S_P producer species are allocated indices of 1 to S_P and the $S - S_P$ consumer species are allocated higher indices, then the rate of change of the biomass of a producer species population *i*, $1 \le i \le S_P$, is given by:

$$\frac{dB_i}{dt} = G_i B_i - \sum_{j=S_P+1}^{S} f_{ij} B_j , \qquad (1.1)$$

where B_i denotes the biomass of a producer species *i*, the coefficient G_i denotes the realised net growth rate of *i* (discounting losses due to metabolism), and f_{ij} is the functional response for consumer species *j* feeding on *i*. Producer species *i* grows at a maximum net growth rate $\sigma_{\max, i}$ in the absence of other producer species and consumption by consumer species. This maximum net growth rate is reduced in the presence of other producer species, due to competition for limiting resources such as light and nutrients and also transmission of diseases (by pathogens such as viruses). The exact form of G_i used for the PDMM version in this study differs from that for the PDMM version used in Fung et al.[26] and is described in detail in the subsection "Detailed description of PDMM: Population dynamics of producer species", together with the underlying rationale. $\sigma_{\max, i}$ scales with the maturation body mass

of i, one of the traits modelled for each species, according to an empirically-derived allometric equation[6].

The functional response f_{ij} is a non-linear Holling type II response extended to include a mechanistic theory of prey-switching among multiple resource species[33]. Functional responses are parameterised by the trophic interaction strengths, c_{kj}, between pairs of species[33]. In the PDMM, these parameters depend on the traits of the interacting species. Specifically, the value of c_{kj} is a product of two factors. The first factor describes the dependence of trophic interaction strengths on the ratio between consumer and resource body masses[8,9,34,35]. The second factor describes the dependence of interaction strengths on the abstract trophic traits of the interacting species. Abstract trophic traits of a species are given by numerical vectors of "foraging" traits and "vulnerability" traits, each of length D, and trophic interactions become stronger when the foraging traits of the consumer j are closer to the vulnerability traits of the resource k [22,24]. Thus, consumption rates are not exclusively determined by the size preference of consumers (preferred ratio between maturation body mass of a consumer species to that of a consumed species), implying that the trophic level of a consumer species does not necessarily increase with its body size (maturation body mass), as observed[8]. The trophic position of a species is defined by all trait values in combination. Specific trait values of species arise from the PDMM assembly algorithm (see subsection "Detailed description of PDMM: Community assembly" for details).

The rate of change of the biomass of a consumer species population i, $S_{\mathrm{P}}+1 \le i \le S$, is given by:

$$\frac{dB_i}{dt} = \varepsilon \sum_{j=1}^{S} f_{ji} B_i - \sum_{j=S_P+1}^{S} f_{ij} B_j - l_{c,i} B_i, \qquad (1.2)$$

where ε is the consumer assimilation efficiency and $l_{c,i}$ is the metabolic loss rate of consumer species *i*. Parameters of the functional response and metabolism are chosen such that populations of consumer species grow at a maximum net rate of $r_{max,i}$ when resources are not limiting and predation is absent. Both $r_{max,i}$ and $l_{c,i}$ scale with the maturation body mass of *i* according to empirically-derived allometric equations[5,36]. In equation (1.2), the first term on the right-hand side represents the rate at which consumer species *i* produces biomass following consumption, and the sum of these terms for all model fish species is used as the measure of production in this study. It is noted that the terms represent the biomass assimilated by fish species and therefore available for human harvesting, which is more directly relevant in the ecosystem service context considered in this study. In other contexts, the biomass ingested may be more appropriate. However, the biomass ingested is simply the biomass assimilated divided by ε. Thus, the results derived in this study for biomass assimilated would also hold if the biomass ingested was used instead, up to a constant scaling factor.

Detailed description of PDMM: Community assembly. In each iteration of the stochastic assembly algorithm of the PDMM, new species are added to the existing model food web, representing invasions. The number of new species is $pS+1$ rounded down to the nearest integer. Here, *p* is a constant and *S* is the number of species in the existing food web. Each new species is a producer or a consumer with equal probability, unless $S = 0$, in which case all new species are producers because only these can survive. The trophic trait values of each new species *k* are determined by varying those of a randomly chosen species *i* in the existing

food web of the same type (producer or consumer), if possible. This introduces phylogenetic correlation of trait values between species (subsection "Overview of PDMM: Community assembly"). Specifically, the maturation body mass of a new species *k*, $M_{\mathrm{mat},k}$, is determined as

$$M_{\mathrm{mat},k} = d^{\xi} M_{\mathrm{mat},i}, \qquad (1.3)$$

where $M_{\mathrm{mat},i}$ is the maturation body mass of species i and ξ is a random number sampled from a standard normal distribution, so that $\log_e^2(d)$ is the variance of $\log_e\left(M_{\mathrm{mat},k}\right)$ when $M_{\mathrm{mat},i}$ is given. If $M_{\mathrm{mat},k}$ falls outside a specified range $\left[M_{\mathrm{min}}, M_{\mathrm{max}}\right]$, a new value is sampled.

The *D* vulnerability trait values for species *k*, arranged in a vector $\mathbf{V}_k$, are determined as

$$\mathbf{V}_k = \mathbf{V}_i + \mu_{\mathrm{V}} \boldsymbol{\xi}, \qquad (1.4)$$

where $\mathbf{V}_i$ is the vector of *D* vulnerability trait values for species *i*, $\boldsymbol{\xi}$ is a vector of independent random numbers sampled from a standard normal distribution, and μ_{V} determines the strength of phylogenetic correlations among vulnerability traits. The vulnerability trait values for both producer and consumer species are constrained within a *D*-dimensional hypersphere (sphere generalised to more than three dimensions) of radius r_{V} and centred at zero. Constraining the values within a hypersphere helps control the volume of trophic niche space and hence species richness[26]. If a newly sampled vector $\mathbf{V}_k$ lies

outside this hypersphere, it is reflected back in at the sphere's surface. If species k is a consumer, then its vector of foraging trait values, $\mathbf{F}_k$, is determined similarly by

$$\mathbf{F}_k = \mathbf{F}_i + \mu_{\mathrm{F}}\boldsymbol{\xi}, \qquad (1.5)$$

where μ_{F} determines the degree of phylogenetic correlations among foraging traits. Analogous to the vulnerability trait values, the foraging trait values are constrained within a hypersphere of radius r_{F} and centered at zero, and if $\mathbf{F}_k$ lies outside this hypersphere, then it is reflected back in at the boundary.

If there are no species in the existing food web of the same type as species k, then the trophic trait values of the latter are assigned default values. Following Fung et al.[26], $M_{\mathrm{mat},k}$ is set to a default value of 10^{-10} kg for producers and a default value of 10^{-7} kg for consumers. In addition, the trait vectors $\mathbf{V}_k$ and $\mathbf{F}_k$ are sampled from the corresponding hyperspheres assuming even probability distributions[26].

Sets of trophic trait values for each new species k are sampled as described above until a set is found giving a positive growth rate (invasion fitness[36]) in the invaded model food web. This leads to a set of new species that can all invade the existing community. These are added to the food web and food-web dynamics with the new species are simulated until an equilibrium is reached or 500 yrs have elapsed. Food-web dynamics are considered to have reached an equilibrium when the biomass dynamics of all species in the food web have reached an equilibrium, according to criteria as described in Fung et al.[26] The waiting time of 500 yrs is longer than the 200 yrs used in Fung et al.[26] and is used to increase the probability that the dynamic response of a model food web to a set of species additions is fully captured.

An equilibrium was reached during the waiting time of 500 yrs for 92% of approximately 1,100 iterations of the assembly algorithm used in the present study.

Detailed description of PDMM: Population dynamics of producer species. Food-web dynamics are specified by a set of differential equations describing the biomass dynamics of each species population. Model dynamics for a producer species *i* is given by equation 1.1 in subsection "Overview of PDMM: Population dynamics and food-web structure". This equation has a term representing growth of the species population, with a net realised growth rate represented by G_i (equation 1.1). Antagonistic interactions between producer species, such as competition or transmission of diseases from one species to the other, are incorporated into G_i. In the previous implementation of the PDMM[26], antagonistic interactions among producer species were controlled by an additional set of abstract traits. In the present PDMM implementation a logistic model for G_i is used instead that allows finer control of producer species richness, following an approach used frequently in food-web models[37–39]. Specifically, producer species *i* grows logistically according to

$$G_i = \sigma_{\max,\, i} \left(1 - \sum_{j=1}^{S_P} \frac{\lambda_{ij} B_j}{K_j} \right), \qquad (1.6)$$

where $\sigma_{\max,\, i}$ is the maximum net growth rate of *i*, λ_{ij} measures the negative effect of producer species *j* on the growth rate of *i* due to competition for limiting resources or transmission of diseases, and K_j is the carrying capacity of *j*. $\sigma_{\max,\, i}$ is given by an allometric equation relating it to the maturation body mass of *i*, following an empirical study[6]:

$$\sigma_{\max, i} = C_{\sigma\max} M_{\mathrm{mat},i}^{\zeta_{\sigma\max}}. \qquad (1.7)$$

The value of λ_{ij} is set to one when $i = j$, reflecting intraspecific competition that is always present, and is otherwise set to a random value that may or may not be zero, reflecting random interspecific antagonistic interactions. Mathematically,

$$\lambda_{ij} = \delta_{ij} + \kappa_{ij}, \qquad (1.8)$$

where $\delta_{ij} = 1$ for $i = j$ and is zero otherwise, and for $i \neq j$ we set $\kappa_{ij} = \kappa$ with probability p_κ and $\kappa_{ij} = 0$ otherwise. With these choices, producer species richness scales approximately as $1/(\kappa^2 p_\kappa)$ [17]. The carrying capacity of species *j*, K_j, is derived from parameters for which empirical data are available, following the approach of previous implementations[23,26]. In a monoculture of producer species *j*, the maximum net rate of biomass production in the model is $\sigma_{\max, j} K_j$. This is set equal to the empirical maximum net rate of biomass production attained by a producer species in monoculture or when dominating a community, which is taken to be a constant based on the observed size-independence in real systems[40]. Thus, $\sigma_{\max, j} K_j = P_{N,\max} a$, where $P_{N,\max}$ is the empirical maximum net rate of biomass production attained by a producer species in monoculture per unit planar area and *a* is the empirical planar area of the system modelled. This gives,

$$K_j = \frac{P_{N,\max} a}{\sigma_{\max, j}}. \qquad (1.9)$$

The loss rate of producer species *i* due to predation by consumer species *j* is determined by the functional response f_{ij}, details of which are presented in the subsection "Detailed description of PDMM: Population dynamics of consumer species" below.

Detailed description of PDMM: Population dynamics of consumer species. The population of consumer species *j* grows by consuming resource species. Consumption of a resource species *i* by a species *j* is specified by an expression for the functional response f_{ij}. This has the same form as in Fung et al.[26] and is given by

$$f_{ij} = \frac{b_j c_{ij} B_i \sum_{k=1}^{S} s_{ik} c_{kj} B_k}{\sum_{k=1}^{S} c_{kj} B_k \left(1 + b_j T_j \sum_{m=1}^{S} s_{km} c_{mj} B_m\right)}, \qquad (1.10)$$

which is a Holling type II response extended to include a mechanistic theory of prey-switching[33]. In this functional response, b_j is the attack rate of consumer species *j*, T_j is the handling time of consumer species *j*, s_{ik} is the similarity between two prey species *i* and *k* with respect to prey-switching, and c_{ij} is the trophic interaction strength between resource species *i* and consumer species *j*. b_j scales allometrically with the maturation body mass of the consumer *j* [7,11,12], $M_{\mathrm{mat},j}$, and can thus be written as

$$b_j = C_b M_{\mathrm{mat},j}^{\zeta_b}. \qquad (1.11)$$

Following Fung et al.[26], T_j can be expressed as

$$T_j = \frac{\varepsilon}{r_{\max,j} + l_{c,j}}, \qquad (1.12)$$

where ε is the consumer assimilation efficiency, $r_{\max,j}$ is the maximum net growth rate of consumer species *j*, and $l_{c,j}$ is the metabolic loss rate of consumer species *j*. Both $r_{\max,j}$ and $l_{c,j}$ scale allometrically with $M_{\mathrm{mat},j}$ (subsection “Overview of PDMM: Population dynamics and food-web structure”):

$$r_{\max,j} = C_{r\max} M_{\mathrm{mat},j}^{\zeta_{r\max}}, \qquad (1.13)$$

$$l_{c,j} = C_{lc} M_{\mathrm{mat},j}^{\zeta_{lc}}. \qquad (1.14)$$

The coefficient s_{ik} is modelled as a decreasing function of the Euclidean distance between the vulnerability trait vectors of the two resource species *i* and *k* [26]. Specifically,

$$s_{ik} = \exp\left(-\frac{|\mathbf{V}_i - \mathbf{V}_k|^2}{2w_s^2}\right), \qquad (1.15)$$

where $1/w_s$ is a measure of the intensity of prey switching. The parameter w_s is called the switching similarity width. As explained in subsection “Overview of PDMM: Population dynamics and food-web structure”, the coefficient c_{ij} depends on the body masses and abstract trophic traits of resource species *i* and consumer species *j*. Specifically[26],

$$c_{ij} = \exp\left(-\frac{|\mathbf{F}_j - \mathbf{V}_i|^2}{2w_c^2}\right)\left[\left(\frac{M_{\mathrm{mat},j}}{M_{\mathrm{mat},i}}\right)\left(\frac{1}{\eta_c}\right)\right]^{-\alpha_c} \quad \text{for} \quad \frac{M_{\mathrm{mat},j}}{M_{\mathrm{mat},i}} > \eta_c, \qquad (1.16a)$$

and

$$c_{ij} = \exp\left(-\frac{|\mathbf{F}_j - \mathbf{V}_i|^2}{2w_c^2}\right)\left[\left(\frac{M_{\mathrm{mat},j}}{M_{\mathrm{mat},i}}\right)\left(\frac{1}{\eta_c}\right)\right]^{\beta_c} \quad \text{for} \quad \frac{M_{\mathrm{mat},j}}{M_{\mathrm{mat},i}} \le \eta_c, \qquad (1.16b)$$

where w_c controls how strongly c_{ij} depends on the proximity of the foraging and vulnerability trait values, and is called the consumer niche width; α_c determines the rate of decrease of c_{ij} when the consumer-resource maturation body mass ratio $M_{\mathrm{mat},j}/M_{\mathrm{mat},i}$ increases above the preferred ratio η_c; and β_c determines the rate of decrease of c_{ij} when $M_{\mathrm{mat},j}/M_{\mathrm{mat},i}$ decreases below η_c. From the subsection "Model parameterisation" below, the empirically derived value of $\eta_c = 10^2$ [8,9]. In addition, following the logic of Fung et al.[26], α_c and β_c were chosen to be 0.07 and 0.25 respectively, such that c_{ij} decreases slowly when the resource species *i* decreases below the preferred maturation body mass of consumer species *j* and decreases quicker when it increases above the preferred mass. These values ensure that consumer species *j* feeds on resource species with a wide range of body masses, which is typical for marine organisms that change their body masses over many orders of magnitude as they grow from larvae to adults (Rossberg[35], Section IX.C). For example, cod (*Gadus morhua*) feed on resource species with a range of maturation body masses spanning more than 7 orders of magnitude, representing small plankton to adult fish[1,3,18,41].

It is noted that the trophic interaction coefficient c_{ij} depends not only on maturation body mass, but also on the foraging traits of consumer species *j* and the vulnerability traits of resource species *i*. Therefore, species *j* does not necessarily feed most on resource species with the preferred body mass, since consumer preferences are also determined by other traits. For example, Supplementary Fig. 1 shows the realised consumer-resource maturation body mass ratio for each fish species in one of the PDMM food webs used, on a logarithmic scale (Model food web 1 in Supplementary Table 3). This ratio is calculated as the weighted geometric mean of the ratios of the maturation body mass of a fish species with the maturation body masses of all its resource species, with the weights being the proportional contribution of each resource species to the diet of the fish species. The ratios for the 206 model fish species approximately span the range 10^{-1}–10^{12}, consistent with empirical body mass ratios found for 1,501 individual pairs of ectotherm vertebrates and their resources[34] (largely covering the range 10^{-2}–10^{14}). This wide range of realised ratios for the model fish species results in a wide range of trophic levels of 2.08-4.99 (Supplementary Table 3), encompassing species that are largely planktivorous to those that are largely piscivorous.

Model parameterisation. Because of the new growth term for producer species, the model implementation used here has the new parameters $P_{N,\max}$, κ, p_κ, $C_{\sigma\max}$ and $\zeta_{\sigma\max}$. This subsection describes how these and other parameters are chosen to achieve realistically complex model marine food webs corresponding to temperate shelf communities in the Northeast Atlantic.

The value of $P_{N,\max}$ is established by the same approach as used for $P_{G,\max}$ (maximum GPP of a single producer species in monoculture per unit planar area) in Fung et al.[26] – i.e., the value is set to lie below upper bounds corresponding to net primary production rates per unit

planar area for the Celtic-Biscay Shelf and North Sea (both situated in the Northeast Atlantic). These upper bounds are 4.99 kg m^{-2} yr^{-1} and 5.81 kg m^{-2} yr^{-1} respectively[4,7], so a value of 0.055 kg m^{-2} yr^{-1} is chosen for $P_{N,\max}$. Furthermore, $C_{\sigma\max}$ and $\zeta_{\sigma\max}$ are set according to the allometric equation in Niklas & Enquist[6] for $\sigma_{\max,i}$, derived using empirical data for unicellular algae. This gives values of 0.182 $kg^{1/4}$ yr^{-1} and -1/4 respectively. The 10 other parameters in the PDMM for which empirical data are available for parameterisation (see Supplementary Table 1) are chosen as in Fung et al.[26], except for a, the system planar area; C_{lc}, the prefactor for the allometric equation defining the metabolic loss rate of consumer species; and η_c, the preferred consumer-resource maturation body mass ratio for consumer species. a is now set to 6×10^{11} m^2, corresponding to the area of the North Sea[4] rather than that of the Celtic Sea. However, following the mathematical argument by Fung et al.[26], changing a has no major effect on model dynamics. C_{lc} is now set to 0.259 $kg^{1/4}$ yr^{-1} rather than 0.298 $kg^{1/4}$ yr^{-1}. The new value is the average of two empirical values from Brown et al.[5] for the metabolic loss rate of consumer species, the first derived using empirical data for fish and the other derived using empirical data for invertebrates, including aquatic types. The value of η_c is set to 10^2 instead of 10^3. Both values are consistent with the empirical range of 10^2-10^3 found using consumer-resource body mass ratios for a demersal fish community[8] and a demersal fish and invertebrate community[9], both in the North Sea.

There are no empirical data to directly constrain κ and p_κ, the remaining two new parameters in the PDMM implementation used here. After all parameters for which empirical data is available had been fixed as described above, the values of these two parameters were varied heuristically together with those of 14 other parameters lacking

empirical data (Supplementary Table 1), until model food webs could reasonably reproduce values of key properties of temperate shelf communities in the Northeast Atlantic (see section “Full details of validation of PDMM food webs” for these properties and corresponding empirical ranges). This heuristic approach follows that in Rossberg et al.[23] and Fung et al.[26]; it is necessary because assembly of each model community is computationally expensive, lasting from days to weeks even with the use of multi-threading over eight processors on a computer cluster. The method gave $\kappa = 0.4$ and $p_{\kappa} = 0.0025$, and values of the other parameters that are the same as in Fung et al.[26], except for $C_b = 1.86 \times 10^{-7}$ kg$^{-3/4}$ yr^{-1}, $d = 2.5$, $r_{\mathrm{V}} = 5.08$, $\mu_{\mathrm{F}} = 1.5$, $\mu_{\mathrm{V}} = 1.4$ and $\alpha_{\mathrm{c}} = 0.07$. Supplementary Table 1 lists all the parameters for the PDMM version used in this study together with the values derived and the corresponding data sources used.

Full details of validation of PDMM food webs

The 20 complex model food webs used in the present study were generated using 20 independent runs of the PDMM assembly algorithm. In each run, the algorithm was stopped at a point where the number of species in the food webs exhibited signs of saturation with the number of species added, representing a balance between speciations and extinctions. Saturation was determined by visual inspection (number of species in a food web approximately constant over at least 2,000 species additions). If the model food web at the end of a run was not at a dynamic equilibrium, then food-web dynamics were simulated for longer until one was reached. In this way, all 20 model food webs used were at a dynamic equilibrium. Any model species with a maturation body mass (M_{mat}) above a minimum

threshold was categorised as a fish species. This minimum threshold for fish species was derived using empirical estimates of M_{mat} for fish species from the Celtic-Biscay Shelf and the North Sea, both situated in the Northeast Atlantic. For fish species in these two regions, M_{mat} was estimated using length at maturity and length-weight regression parameter values from FishBase[3]. It was found that >96% (194/201) and >95% (168/176) of fish species in the Celtic-Biscay Shelf and the North Sea, respectively, have $M_{\text{mat}} > 10^{-3}$ kg. Thus, the minimum threshold was set to 10^{-3} kg. Supplementary Fig. 2 is a graphical representation of one of the 20 model food webs produced, showing the biomass density and M_{mat} of each species in the web.

There is the issue of whether non-fish species largely have maturation body masses below the 10^{-3} kg threshold. Robinson et al.[42] sampled 178 benthic invertebrate species from the North Sea – including crustaceans, echinoderms, molluscs and polychaetes – and derived a range of body masses for each sampled species. They did not derive the maturation body masses, but if the maximum body masses are assumed to be approximately equal to the asymptotic body masses and equations relating maturation body mass and asymptotic body mass are assumed to be the same as for marine fish (these equations are given below in the description of how the slopes of the diversity spectra for model food webs were calculated), then 46/178 (26%) species have $M_{\text{mat}} > 10^{-3}$ kg. However, a relatively small 18/178 (10%) of species have $M_{\text{mat}} > 10^{-2}$ kg, suggesting that most of the true M_{mat} values are near the 10^{-3} kg threshold. Thus, it is conceivable that a 10^{-3} kg threshold largely differentiates non-fish from fish species. Cephalopods and scyphozoans can also have a maturation body mass above 10^{-3} kg. However, the most recent data suggest that their diversity is much lower than that of marine fish – there are approximately 700 species worldwide compared to

approximately 15,000 marine fish species[19]. Thus, exclusion of cephalopod and scyphozoan species with $M_{\mathrm{mat}} > 10^{-3}$ kg in the PDMM is not likely to have a major effect on the BEF relations derived. In addition, species above $M_{\mathrm{mat}} > 10^{-3}$ kg in the PDMM can be re-interpreted as not just fish species, but those species that are fished. In this case, it would not matter whether there is a relatively small proportion of non-fish species with $M_{\mathrm{mat}} > 10^{-3}$ kg.

Details of food-web properties used for model validation. Following Fung et al.[26], each of the 20 PDMM food webs was validated by comparing values of key food-web properties with empirically derived values for real temperate shelf communities. The latter were derived using empirical data from the Northeast Atlantic where possible. In total, 10 food-web properties were examined. The first six properties are the same as in Fung et al.[26] These are (i) phytoplankton species richness, (ii) fish species richness, (iii) the dietary diversity of fish species, (iv) the range of maturation body masses for phytoplankton species, (v) the range of maturation body masses for fish species, and (vi) the range of trophic levels for fish species. Dietary diversity (iii) is defined as the average number of resource species consumed by a fish species, counting all resource species that contribute >1% to the diet of a fish species[43]. For (vi), the trophic level for a fish species was calculated as 1 plus the weighted average trophic level of the species that it consumes, with weights given by the biomass intake rates normalised by the total biomass intake rate, i.e. proportional contributions to diets. For the present study, these six properties were supplemented by four more properties: (vii) the diet-partitioning exponent, which is estimated as the slope of a linear regression of the logarithm of the dietary diversity of fish species against the logit of the proportional diet contribution above which a consumed species is counted as a resource[16,17]; (viii) the slope of the diversity spectrum, which is the slope of a fitted probability density function of asymptotic body masses of fish species on a $\log_{10}$-$\log_{10}$

scale[19]; (ix) the slope of the biomass size-spectrum, which is the slope of a linear regression of biomass against individual body mass for fish species on a $\log_{10}$-$\log_{10}$ scale[20]; and (x) the range of biomass densities for fish species. Use of (vii), (viii) and (ix) allowed more rigorous quantitative assessments of trophic link structure, size-structure among species and size-structure among individuals, respectively. Use of (x) allowed a quantitative assessment of fish abundances. Together, the 10 food-web properties examined are major determinants of food-web structure and dynamics.

In calculating the slope of the diversity spectrum for a model food web, the method detailed in Section S10.2 of the Online Appendix of Reuman et al.[19] was used. This involved fitting the cumulative distribution function (cdf) of a truncated Pareto distribution to the cdf of asymptotic body masses (M_∞) of all model fish species, using maximum likelihood estimates of the Pareto distribution parameters[44]. The maturation body masses (M_{mat}) of the model fish species were converted to M_∞ by first using the equation $\log_{10}(L_{\max}) = 0.342\log_{10}(M_{\text{mat}}) + 1.93$ to convert M_{mat} (in kg) to $L_{\max}$ (in cm), the maximum length of a fish species. This equation is derived by performing a linear regression on pairs of M_{mat} and $L_{\max}$ values for 118 fish species from the North Sea (same data as used in Fung et al.[21]; $R^2 = 0.580$). Then assuming that $L_{\max} = L_\infty$, following Reuman et al.[19], the equation $\log_{10}(M_\infty) = 2.54\log_{10}(L_\infty) - 4.044$ is used to convert L_∞ to M_∞ (in kg). This equation was derived in Reuman et al.[19] using data from 525 fish species records from the International Game Fish Association ($R^2 = 0.877$). Goodness-of-fit of the cdf of the truncated Pareto distribution to the cdf of the M_∞ values was assessed using the coefficient of determination (R^2), following Reuman et al.[19] The slope of the diversity spectrum is derived as the value of

the shape parameter for the truncated Pareto distribution minus one, following calculations in Reuman et al.[19] (Section S10.2 of their Online Appendix).

To calculate the slope of the biomass size-spectrum of a model food web, the graph of biomass against individual body mass for the model fish species was drawn first, on a $\log_{10}$-$\log_{10}$ scale. Since the PDMM does not resolve the intraspecific size-structure of model species, we assumed that each model fish species at equilibrium has an intraspecific size-structure of the form derived by Rossberg[35] (equation [114]) using a size-structured fish community model. Once the graph was drawn, a linear regression over the body mass range 0.1-10 kg was performed to derive the slope. This range is chosen because it allows direct comparison of slopes with the empirically derived values from Jennings & Blanchard[20].

Empirical values for (vii) were taken from Rossberg et al.[16] and pertain to the same locations as empirical values for (iii), whereas empirical values for (viii) were taken from the analyses of Reuman et al.[19] for the fish communities in the Celtic-Biscay Shelf and North Sea. Empirically derived values for (xi) were taken from Jennings & Blanchard[20], who applied macroecological theory to trawl survey data for fish in the North Sea. Empirical values for (x) were taken from the analysis of Fung et al.[21] for the North Sea demersal fish community. Data sources and study locations pertaining to the empirically derived values for all 10 properties are summarised in Supplementary Table 2.

Detailed model validation results. We assessed the realism of our model food webs by comparing values of their structural properties with those of the natural communities represented. Supplementary Tables 3-6 show values of the 10 properties tested for each of the 20 model food webs together with empirically derived values for temperate shelf

communities. These tables supplement Table 1 in the main text, which showed a comparison of model versus empirically derived values considering all the 20 model food webs as a group.

Fish species richness of nine model food webs were within the empirical range, with richness for the remaining 11 webs being within 23% of the empirical range (Table 1; Supplementary Tables 3-6). Model phytoplankton species richness consistently exceeded the highest empirical estimate, by 51-74% (Table 1; Supplementary Tables 3-6). The dietary diversity of fish species[16] quantifies the trophic link density among fish species, and thus implicitly quantifies their trophic niche widths. Model values for this property fell within the empirical range (Tables 1; Supplementary Tables 3-6). In addition, model values of the diet partitioning exponent[16], which reflects the distribution of trophic link strengths, also fell within the corresponding empirical range (Table 1; Supplementary Tables 3-6). Supplementary Fig. 3 graphically depicts the relationship between dietary diversity and diet threshold for 10 of the 20 model food webs. In every web, the maturation body masses of all phytoplankton and fish species fell within the empirical ranges (Table 1; Supplementary Tables 3-6). Furthermore, >86.2% of fish species in any one food web had a trophic level within the empirical range, whereas >96.4% of fish species had a biomass density within the empirical range (Table 1; Supplementary Tables 3-6). Moreover, in every web, the maturation body masses, trophic levels and biomass densities described cover 54.6-100% of the corresponding four empirical ranges (using a $\log_{10}$ scale for body masses and biomass densities), except the biomass densities for one web, which cover 41.3% of the corresponding empirical range (Table 1; Supplementary Tables 3-6).

To further characterise the distribution of species among different size classes, we calculated the slope of the diversity spectrum for each model food web[19]. Using a lower asymptotic body mass bound of 1 kg [19] (0 on a $\log_{10}$ scale), slopes for 11 food webs fell within the empirical range, whereas slopes for five food webs were within 9% of the empirical range (Table 1; Supplementary Tables 3-6). Slopes for the remaining four webs (0.0000976-0.654) fell outside the empirical range, but fell within the range when the lower asymptotic body mass bound was increased to 3-35 kg (0.477-1.54 on a $\log_{10}$ scale; Table 1; Supplementary Tables 3-6). All fitted functions (considering a 3-35 kg lower asymptotic body mass bound for the four webs mentioned) gave a good fit to the underlying data from the model food webs, with $R^2 > 0.92$ (Supplementary Fig. 4 shows the diversity spectrum for one of the 20 PDMM food webs together with the fitted spectrum). We also estimated the slope of the biomass size-spectrum for each model food web[20]. Considering the body mass range of 0.1-10 kg [20] (-1 to 1 on a $\log_{10}$ scale), slopes for 16 food webs fell within the expected range predicted by applying macroecological theory to empirical data, with slopes for the remaining four webs being more negative by 9-114% (Table 1; Supplementary Tables 3-6; Supplementary Fig. 5 shows the biomass size-spectra for 10 of the PDMM food webs). The linear regressions were generally a good fit to the underlying data from the model food webs, with $R^2 > 0.67$ for 12 webs; however, they gave poor fits for eight webs, with $R^2 < 0.14$. It is noted that the biomass size-spectra for the model webs all exhibit a declining or approximately linear trend, such that total biomass of large individuals tends to be no greater than that of smaller individuals. This does not contradict the slight increasing trends observed for species population biomass with maturation body mass, since there are typically fewer species at large maturation body masses (Supplementary Fig. 2).

Interpretation of model validation results. Food-web structure can be characterised by the number, biomasses, body sizes and trophic link strengths of constituent species[45,46]. Together, these structural elements are fundamental determinants of energy flows between species, so reproducing these elements is expected to capture essential aspects of food-web dynamics and stability. We compared values of 10 properties quantifying these elements for the 20 complex model food webs used with empirically derived values from temperate shelf communities. There was largely good agreement, allowing the model webs to be taken as realistically representing the food-web structure of temperate shelf communities. Although model phytoplankton species richness exceeded the highest empirical estimate by 51-74% (Table 1; Supplementary Tables 3-6), empirical counts are likely to be underestimates because of incomplete sampling and identification[47]. The slopes of the model biomass size-spectra were largely within the range derived by applying macroecological theory to empirical data[20], but the corresponding linear regressions used to calculate the slopes often gave poor fits to the model spectra. This could be because of the presence of food-web effects independent of body size in the PDMM[23], which introduce extra variation not captured in macroecological theory[20].

The realistic structure of the model food webs is expected to give realistic production values. Indeed, Heath[48] estimated that fish in the North Sea consume 13.9-19.5 gC m^{-2} yr^{-1} over the period 1973-1999. Using conversion factors of 1 gC = 1/0.4 g dry weight[7] and 1 g dry weight = 1/0.32 g wet weight[49], and assuming an assimilation efficiency of 0.6 [10,50], total fish production in the North Sea was 65.2-91.4 g m^{-2} yr^{-1}. This is in good agreement with the mean value of 96.6 g m^{-2} yr^{-1} for the model webs, especially considering that the empirical range could be an underestimate due to historical fishing[20].

Partitioning variation in production

By performing random deletion experiments with 20 PDMM food webs, the relationship between (normalised) fish species richness and mean total fish biomass production was derived in the main text, together with the standard errors in this production (Fig. 1). The standard error at a particular richness value measures the variation in the mean production, not in the production values. Thus, in Supplementary Fig. 10, we show the mean relationship together with the standard deviations in production. This variation in production exhibits an increasing trend with richness. However, the coefficient of variation remains largely similar across the entire range of richness values (Supplementary Fig. 8).

In order to obtain a deeper insight into the sources of variation, the variance in production was partitioned according to variance between food webs and within food webs. This was done by considering each fish species richness value in turn, calculating the sum of squared differences between all production values and the mean production for the species richness value considered, and then partitioning this sum into contributions due to differences between and within webs. More precisely, at a particular richness value of $\Phi = \Phi_x$, denote the number of corresponding production values produced from the 10 random deletion experiments for PDMM food web i ($1 \le i \le 20$) by $n_{x,i}$ and the production values by $P_{x,ij}$ ($1 \le j \le n_{x,i}$). Then the sum of squared differences is

$$SS_x = \sum_{i=1}^{20} \sum_{j=1}^{n_{x,i}} \left(P_{x,ij} - \bar{P}_{x,\cdot\cdot} \right)^2 , \qquad (3.1)$$

where $\bar{P}_{x,\cdot\cdot}$ is the mean over all *i* and *j*. The contribution to this sum due to variation in production between food webs is calculated as

$$SS_{x,\mathrm{b}} = \sum_{i=1}^{20} \left(\bar{P}_{x,i\cdot} - \bar{P}_{x,\cdot\cdot} \right)^2 , \qquad (3.2)$$

where $\bar{P}_{x,i\cdot}$ is the mean over all *j* for given *i*. The contribution to the sum due to variation in production within food webs (arising from use of 10 random deletion experiments for each web) is calculated as

$$SS_{x,\mathrm{w}} = \sum_{i=1}^{20} \sum_{j=1}^{n_{x,i}} \left(P_{x,ij} - \bar{P}_{x,i\cdot} \right)^2 . \qquad (3.3)$$

Thus, $SS_x = SS_{x,\mathrm{b}} + SS_{x,\mathrm{w}}$. Supplementary Fig. 9 shows that $SS_{x,\mathrm{b}}$ dominates $SS_{x,\mathrm{w}}$ for a large range of Φ (approximately $\Phi > 0.1$) and tends to increase with Φ. This shows that increasing variation in production is due predominantly to greater differences between food webs rather than differences within food webs.

Mathematical analyses of BEF relations

Mean-field theory. In a Lotka-Volterra model describing the food-web dynamics of *S* fish species, the rate of change of the biomass density of species *i* in kg m^{-2}, ρ_i, is given by:

$$\frac{d\rho_i}{dt} = \rho_i \left(r_i - \sum_{j=1}^{S} \alpha_{ij} \rho_j \right), \qquad (4.1)$$

where r_i is the intrinsic population growth rate of species *i* in yr^{-1} and α_{ij} is the interaction coefficient quantifying the effect of species *j* on *i* in kg^{-1} m^2 yr^{-1}. r_i represents gains in the biomass of fish species *i* because of consumption of non-fish species minus losses due to non-predation processes, largely metabolism. We first examine the general case where the interspecific interaction coefficients can be different from each other, such that α_{ij} does not have to be the same as α_{ij}. This general case encompasses food webs with their asymmetric interactions. In the subsection "Implications of competition symmetry" below, we will examine the special case where the interspecific interaction coefficients are symmetric, i.e. $\alpha_{ij} = \alpha_{ji}$, in order to provide a contrast to results for the general case that we derive in this subsection. The symmetric case pertains to species that partake predominantly in competitive rather than trophic interactions.

In the general case, we apply a mean-field approximation, under which it is assumed that interspecific interaction strengths are largely independent, such that $\left\langle \sum_{j=1}^{S} \alpha_{ij} \rho_j \right\rangle \approx S \langle \rho_i \rangle \langle \alpha_{ij} \rangle$. Here, the angled brackets denote expectation values for random choices of quantities with unsummed indices inside the brackets[51,52]. When evaluating the expected interaction coefficient, $\langle \alpha_{ij} \rangle$, there is a need to distinguish between the expected contribution from off-diagonal terms, $\langle \alpha_{ij} \rangle_{i \neq j}$, and the expected contribution from diagonal terms, $\langle \alpha_{ii} \rangle$, because the latter tends to be systematically larger than the former. This gives $\langle \alpha_{ij} \rangle = \left[(S-1) \langle \alpha_{ij} \rangle_{i \neq j} + \langle \alpha_{ii} \rangle \right] / S$. The resulting model is simpler compared with the PDMM

(section "Further details on the Population-Dynamical Matching Model (PDMM)"), notably by having linear rather than non-linear functional responses and by following Wilson et al.[52], Wilson & Lundberg[53] and Bastolla et al.[54] (among others) in applying the mean-field approximation. The purpose of examining this model is to determine whether a simpler, analytical model is able to reproduce the same BEF relations as the PDMM. This provides an insight into whether the simplifying assumptions in the simpler model can be made without affecting BEF predictions.

Using the mean-field approximation, the equilibrium conditions for the dynamic system specified by equation 4.1 can be evaluated to give

$$\langle \rho_i \rangle = \frac{\langle r_i \rangle}{(S-1)\langle \alpha_{ij} \rangle_{i \neq j} + \langle \alpha_{ii} \rangle} \qquad (4.2)$$

[51,52]. The mean total biomass density, $\bar{\rho}$, can then be obtained as $\bar{\rho} = S\langle \rho_i \rangle$. It is assumed that the mean total biomass production per unit area, $\overline{P}$, is equal to $\bar{\rho}$ multiplied by a constant C with units of yr^{-1}. Under this assumption, the mean-field approximation gives

$$\overline{P} = CS\frac{\langle r_i \rangle}{(S-1)\langle \alpha_{ij} \rangle_{i \neq j} + \langle \alpha_{ii} \rangle}. \qquad (4.3)$$

Denoting the number of fish species in the pristine (unfished) state as S_{pris}, the equation for $\overline{P}$ can be rearranged to give a Michaelis-Menten (MM) function:

$$\overline{P} = \frac{A\Phi}{\Phi + B}, \qquad (4.4)$$

where $\Phi = S/S_{\text{pris}}$ is the normalised fish species richness, $A = C\langle r_i \rangle / \langle \alpha_{ij} \rangle_{i \neq j}$ and

$B = \left(\langle \alpha_{ii} \rangle - \langle \alpha_{ij} \rangle_{i \neq j} \right) / \left(S_{pris} \langle \alpha_{ij} \rangle_{i \neq j} \right)$.

$\langle r_i \rangle$, $\langle \alpha_{ij} \rangle_{i \neq j}$ and $\langle \alpha_{ii} \rangle$ can be set to achieve values of *A* and *B* matching that for the MM function fitted to PDMM simulation results, which are 0.154 kg m^{-2} yr^{-1} and 0.533 respectively (Fig. 1b in main text). Using $S_{\text{pris}} = 190$, which is the average number of fish species in the 20 PDMM food webs used, and $C = 0.745$ yr^{-1}, which is calculated using a linear regression of $\overline{P}$ and $\overline{\rho}$ derived using the 20 food webs ($n = 300, r^2 = 0.977$), the two equations for *A* and *B* contain the three unknowns $\langle r_i \rangle$, $\langle \alpha_{ij} \rangle_{i \neq j}$ and $\langle \alpha_{ii} \rangle$. The equation for *B* can be solved to obtain the ratio $\langle \alpha_{ij} \rangle_{i \neq j} / \langle \alpha_{ii} \rangle = 0.00978$, which measures the average strength of interspecific interactions relative to that of intraspecific interactions among fish. Using this ratio in the equation for *A* then gives $\langle \alpha_{ii} \rangle = 495 \langle r_i \rangle$ kg^{-1} m^2. Although we do not derive an estimate of $\langle r_i \rangle > 0$ here, it is clear that however $\langle r_i \rangle$ is chosen, there are positive values of $\langle \alpha_{ii} \rangle = 495 \langle r_i \rangle$ kg^{-1} m^2 and $\langle \alpha_{ij} \rangle_{i \neq j} = 0.00978 \langle \alpha_{ii} \rangle$ that give a MM function which matches the one fitted to the PDMM simulation results. Biologically, $\langle r_i \rangle$ represents the expected net effect of gains in biomass of fish species *i* due to feeding on non-fish species and non-predation losses due largely to metabolism. Thus, $\langle r_i \rangle > 0$ means that a fish species typically has a positive net growth rate in the absence of other fish species. This does not necessarily mean that r_i is positive for all fish species – for example, largely piscivorous fish

species are likely to have $r_i < 0$. It only means that the average r_i is positive. This is consistent with the fact that 81% of the 3,805 fish species in the 20 PDMM food webs have a positive net growth rate in the absence of all other fish species. Similarly, the condition $\langle \alpha_{ij} \rangle_{i \neq j} = 0.00978 \langle \alpha_{ii} \rangle$ implies that the average interspecific interaction strength between two fish species is smaller than the average intraspecific interaction strength within a fish species. This does not preclude a subset of the interspecific interaction strengths for a particular fish species being greater than the intraspecific interaction strength for that species, which may be the case for largely predatory fish species.

To understand how $\overline{P}$ changes when a fish species is randomly deleted, consider the change in $\overline{P}$, $\Delta\overline{P}$, when a fish species is randomly deleted from a food web with *S* fish species. With this deletion, *S* changes by $\Delta S = -1 - s$, where *s* is the rate of secondary extinctions of fish species. Thus, $\Delta\overline{P}$ can be approximated by

$$\Delta\overline{P} = \frac{d\overline{P}}{dS}\Delta S = -\frac{d\overline{P}}{dS}\left(1+s\right). \qquad (4.5)$$

$-d\overline{P}/dS$ can be calculated by writing $\overline{P}$ as a function of *S* and differentiating with respect to *S*:

$$\overline{P} = \frac{A\left(S/S_{\text{pris}}\right)}{\left(S/S_{\text{pris}}\right)+B} = \frac{AS}{S+BS_{\text{pris}}}, \qquad (4.6)$$

$$-\frac{d\overline{P}}{dS} = -\frac{A}{S+BS_{\text{pris}}} + \frac{AS}{\left(S+BS_{\text{pris}}\right)^2}. \qquad (4.7)$$

Thus,

$$\Delta\overline{P} = \left(-\frac{A}{S + BS_{\text{pris}}} + \frac{AS}{\left(S + BS_{\text{pris}}\right)^2} \right)(1+s). \qquad (4.8)$$

In random-deletion simulations using 20 PDMM food webs (10 replicates per web), the average *s* across all replicates and food webs is 0.26, which is small. Thus, in equation 4.8, *s* can be taken to be 0 as a simplifying approximation, which gives

$$\Delta\overline{P} = -\frac{A}{S + BS_{\text{pris}}} + \frac{AS}{\left(S + BS_{\text{pris}}\right)^2}. \qquad (4.9)$$

The first term on the right of $\Delta\overline{P}$ equals $-\overline{P}/S$, which is the expected immediate loss in *P* due to random deletion of a fish species. Therefore, the second term on the right must represent the expected change in *P* due to dynamic responses following random deletion of a fish species. This can be verified by observing that the second term is equal to $-d\left(\overline{P}/S\right)/dS = A/\left(S + BS_{\text{pris}}\right)^2$ multiplied by *S*. $-d\left(\overline{P}/S\right)/dS = A/\left(S + BS_{\text{pris}}\right)^2$ represents the expected change in production of a fish species resulting from random deletion of another fish species. Thus, $-d\left(\overline{P}/S\right)/dS$ multiplied by *S* represents the expected change in the total production of all remaining fish species following the random deletion, which is the expected change in *P* due to dynamic responses following the deletion. In Fig. 2a in the main text, the two terms of equation 4.9 are plotted as functions of normalised fish species richness, Φ. To achieve this, $\Delta\overline{P}$ is rewritten as

$$\Delta\overline{P} = -\frac{A}{S_{\text{pris}}}\frac{1}{\Phi + B} + \frac{A}{S_{\text{pris}}}\frac{\Phi}{(\Phi + B)^2}. \qquad (4.10)$$

The second term, corresponding to the effect of dynamic responses on *P*, is consistently higher by small amounts than simulation results using the 20 PDMM food webs. This discrepancy is a consequence of the approximation of disregarding secondary extinctions ($s = 0$) in the derivation of the second term. When including this effect in the derivation (using equation 4.8), the corresponding correction to the second term is

$$-\frac{A}{S + BS_{\text{pris}}}\left(1 - \frac{S}{S + BS_{\text{pris}}}\right)s, \qquad (4.11)$$

which is always negative for $A, B > 0$. The precise value is difficult to predict, because *s* depends on *S*.

Implications of competition symmetry. This subsection recalls a few results on the implications of interaction symmetry for richness-production relations, to contrast the results derived in subsection "Mean-field theory" for the more general case where interactions do not have to be symmetric. The argument is again based on the generalised Lotka-Volterra model, here interpreted as describing the effective dynamics and interactions within a fish community. It is noted that the Lotka-Volterra model is a simplified, coarse approximation of the true dynamics of communities. Nevertheless, it is often used in qualitative analyses. In essence, the results summarised here were already encapsulated in MacArthur's niche

theory[55,56] (see Chapter 2 of Loreau[57] for a detailed synthesis). The presentation here closely follows and expands on Rossberg[17].

Define

$$Q = 2\sum_{i=1}^{S} \rho_i r_i - \sum_{i=1}^{S}\sum_{j=1}^{S} \rho_i \alpha_{ij} \rho_j \ , \qquad (4.12)$$

with symbols as in equation 4.1. Q is defined in this way because at a community equilibrium, it simplifies to a measure of fish community biomass production, as will be shown below. First, by computing the time derivative of Q and making use of equation 4.1, one obtains

$$\frac{dQ}{dt} = \sum_{i=1}^{S} \rho_i \left(r_i - \sum_{j=1}^{S} \alpha_{ij}\rho_j \right)\left(2r_i - \sum_{j=1}^{S} \alpha_{ij}\rho_j - \sum_{j=1}^{S} \rho_j \alpha_{ji} \right) . \qquad (4.13)$$

With symmetric interactions, that is $\alpha_{ij} = \alpha_{ji}$, the time derivative simplifies to

$$\frac{dQ}{dt} = 2\sum_{i=1}^{S} \rho_i \left(r_i - \sum_{j=1}^{S} \alpha_{ij}\rho_j \right)^2 \ , \qquad (4.14)$$

which is always non-negative and zero only when an equilibrium has been reached. The value of Q therefore steadily increases until an equilibrium is reached. For arbitrary asymmetric interaction matrices this is generally *not* the case. The symmetry $\alpha_{ij} = \alpha_{ji}$ shall be assumed henceforth.

From equation 4.12 one can see that, when S is replaced by $S+1$ and a new species $k=S+1$ is added to the system at very low abundance ($\rho_k \approx 0$, $\rho_k > 0$), the value of *Q* essentially does not change. Similarly, removal of a species that has reached very low abundance essentially does not change the value of *Q*. The value of *Q* therefore increases steadily during modelled community assembly processes in which new species are added at abundance close to zero and species are being removed if their abundances decline to values close to zero.

Whenever equilibria are reached during such assembly processes, one can make use of the equilibrium condition $\rho_i r_i = \sum_{j=1}^{S} \rho_i \alpha_{ij} \rho_j$ (for all *i*) to simplify equation 4.12 for *Q* to

$$Q = \sum_{i=1}^{S} \rho_i r_i \qquad \text{(at equilibrium).} \qquad (4.15)$$

It follows that $\sum_{i=1}^{S} \rho_i r_i$ increases from one equilibrium state to another during a modelled community assembly process, provided that interactions are symmetric.

Assume the community has reached a locally asymptotically stable equilibrium with *S* species. How will *Q* change if a subset of the *S* species in the community is selected and deleted from the community, and populations are allowed to relax until a new equilibrium is reached? To answer this question, call the original *S*-species equilibrium community the *high-diversity community*. Denote as the *low-diversity community* the community at the new equilibrium after species deletion – this community is obtained from the *high-diversity*

community by deleting the subset of selected species as well as any species that reach zero abundance during dynamics to the new equilibrium (secondary extinctions). Because α_{ij} is symmetric, the local asymptotic stability of the high-diversity community implies its global stability.* Thus, if one re-inserts all species deleted from the high-diversity community into the low-diversity community at low abundances, the original high-diversity community will re-emerge. By the considerations above, the value *Q* will increase in this process. This implies directly that *Q* is lower for the low-diversity community than it is for the high-diversity community. Hence, for symmetric interactions, the equilibrium value of $\sum_{i=1}^{S} \rho_i r_i$ declines with any species deletion.

Since ρ_i is the biomass density of species *i*, one can interpret $\sum_{i=1}^{S} \rho_i r_i$ as a measure of total community production. This will generally be different from the measure of total system production used in the main text. The Lotka-Volterra model is simply too abstract to isolate gross production among the various biological contributions to population dynamics. On the other hand, the approximation that production is proportional to biomass for a given species (constant production/biomass ratio or turnover rate) is frequently applied in the ecological

---

* Local stability of the system at an inner ($\rho_i > 0$) equilibrium point implies that the Jacobian matrix at equilibrium with entries $J_{ij} = -\rho_i \alpha_{ij}$ has no eigenvalues with positive real parts. The eigenvalues of the Jacobian $\mathbf{J}$ are the same as the eigenvalues of the real, symmetric matrix with entries $\rho_i^{-1/2} J_{ij} \rho_i^{1/2} = -\rho_i^{1/2} \alpha_{ij} \rho_i^{1/2}$. All eigenvalues of this matrix are real. By the stability of the equilibrium point they are ≤ 0. From Sylvester's law of inertia[58] it then follows that the matrix $-\boldsymbol{\alpha}$ has the same number of negative and zero eigenvalues as $\mathbf{J}$, and no positive eigenvalues. Now, consider first the case that zero is an eigenvalue of $\boldsymbol{\alpha}$, and let $\mathbf{u}$ be a vector in the null space of $\boldsymbol{\alpha}$. It is then not difficult to verify that with any equilibrium ρ_i of the Lotka-Volterra system and any real number ε, the point $\rho_i + \varepsilon u_i$ is an equilibrium as well. So ρ_i is not locally asymptotically stable in this case, in violation of the premise. This implies that all eigenvalues of $-\boldsymbol{\alpha}$ are negative, so that $-\boldsymbol{\alpha}$ is negative definite. According to a theorem by Getz[59], the fixed point is then a global attractor for any initial condition with positive abundances for all species.

literature (e.g. Brey & Clarke[60], Epstein, et al.[61] and Jennings et al.[62]), justifying the interpretation of $\sum_{i=1}^{S} \rho_i r_i$ as a proxy for total community production.

When applying these results to deduce generalisations about BEF relations, two caveats need to be kept in mind: (i) even for purely competitive interactions, perfect symmetry of the interaction matrix cannot be expected and (ii) the Lotka-Volterra model provides just a coarse approximation of the dynamics of an ecological community. Therefore, what we have demonstrated for symmetric Lotka-Volterra systems should only be interpreted as a general rule for natural communities dominated by competition – i.e. there will be exceptions. Despite these caveats, however, we have shown that interaction symmetry is essential for obtaining a steady increase of production with each species added to a community and a steady decrease of production with each species deleted, where in both cases secondary extinctions might occur as a result of the manipulations. For strongly asymmetric interactions, this cannot be expected. However, as detailed in the subsection "Mean-field theory" above, in the specific case of random additions and deletions of species, these trends can still be expected to be seen on average. Thus, the key difference between the general case where interactions can be asymmetric and the specific case where interactions are symmetric is that in the former case, production increases *on average* with increasing richness, whereas in the latter case, production always increases with increasing richness.

Supplementary References

1. Beardall, J. B. *et al*. Allometry and stoichiometry of unicellular, colonial and multicellular phytoplankton. *New Phytol*. **181**, 295–309 (2009).

2. Fenchel, T. & Finlay, B. J. Respiration rates in heterotrophic, free-living protozoa. *Microb. Ecol*. **9**, 99–122 (1983).

3. Froese, R. & Pauly, D. *Fishbase version (09/2010)*. http://www.fishbase.org (2010).

4. Sea Around Us Project. *Sea Around Us Project: Large Marine Ecosystems*. http://www.seaaroundus.org/lme (2010).

5. Brown, J. H., Gillooly, J. F., Allen, A. P., Savage, V. M. & West, G. B. Toward a metabolic theory of ecology. *Ecology* **85**, 1771–1789 (2004).

6. Niklas, K. J. & Enquist, B. J. Invariant scaling relationships for interspecific plant biomass production rates and body size. *Proc. Natl. Acad. Sci. USA* **98**, 2922–2927 (2001).

7. Peters, R. H. *The Ecological Implications of Body Size* (Cambridge University Press, Cambridge, 1983).

8. Jennings, S., Pinnegar, J. K., Polunin, N. V. C. & Boon, T. W. Weak cross-species relationships between body size and trophic level belie powerful size-based trophic

structuring in fish communities. *J. Anim. Ecol.* **70**, 934–944 (2001).

9. Jennings, S., Pinnegar, J. K., Polunin, N. V. C. & Warr, K. J. Linking size-based and trophic analyses of benthic community structure. *Mar. Ecol. Prog. Ser.* **226**, 77–85 (2002).

10. Hendriks, A. J. The power of size: a meta-analysis reveals consistency of allometric regressions. *Ecological Modelling* **205**, 196–208 (2007).

11. Ware, D. M. Bioenergetics of pelagic fish: theoretical change in swimming speed and ration with body size. *Journal of the Fisheries Research Board of Canada* **35**, 220–228 (1978).

12. Andersen, K. H. & Beyer, J. E. Asymptotic size determines species abundance in the marine size spectrum. *Am. Nat.* **168**, 54–61 (2006).

13. Savage, V. M., Gillooly, J. F., Brown, J. H., West, G. B. & Charnov, E. L. Effects of body size and temperature on population growth. *Am. Nat.* **163**, 429–441 (2004).

14. Smith, V. H. *et al.* Phytoplankton species richness scales consistently from laboratory microcosms to the world's oceans. *Proc. Natl. Acad. Sci. USA* **102**, 4393–4396 (2005).

15. Ojaveer, H. *et al.* Status of biodiversity in the Baltic Sea. *PLOS ONE* **5**, e12467 (2010). doi:10.1371/journal.pone.0012467.

16. Rossberg, A. G., Farnsworth, K. D., Satoh, K. & Pinnegar, J. K. Universal power-law diet partitioning by marine fish and squid with surprising stability-diversity implications. *Proc. R. Soc. B* **278**, 1617–1625 (2011).

17. Rossberg A. G. *Food Webs and Biodiversity: Foundations, Models, Data* (Wiley, Hoboken, 2013).

18. Barnes, C., Irigoien, X., de Oliveira, J. A. A., Maxwell, D. & Jennings, S. Predicting marine phytoplankton community size structure from empirical relationships with remotely sensed variables. *J. Plankton Res.* **33**, 13–24 (2011).

19. Reuman, D. C., Gislason, H., Barnes, C., Mélin, F. & Jennings, S. The marine diversity spectrum. *J. Anim. Ecol.* **83**, 963–979 (2014).

20. Jennings, S. & Blanchard, J. L. Fish abundance with no fishing: predictions based on macroecological theory. *J. Anim. Ecol.* **73**, 632-642 (2004).

21. Fung, T., Farnsworth, K. D., Reid, D. G. & Rossberg, A. G. Recent data suggest no further recovery in North Sea Large Fish Indicator. *ICES J. Mar. Sci.* **69**, 235–239 (2012).

22. Rossberg, A. G., Matsuda, H., Amemiya, T. & Itoh, K. Food webs: Experts consuming families of experts. *J. Theor. Biol.* **241**, 552–563 (2006).

23. Rossberg, A. G., Ishii, R., Amemiya, T. & Itoh, K. The top-down mechanism for body-

mass–abundance scaling. *Ecology* **89**, 567–580 (2008).

24. Rossberg, A. G., Brännström, Å. & Dieckmann, U. How trophic interaction strength depends on traits: A conceptual framework for representing multidimensional trophic niche spaces. *Theoretical Ecology* **3**, 13–24 (2010).

25. Shephard, S. *et al*. Size-selective fishing drives species composition in the Celtic Sea. *ICES J. Mar. Sci.* **69**, 223–234 (2012).

26. Fung, T., Farnsworth, K. D., Shephard, S., Reid, D. G. & Rossberg A. G. Why the size structure of marine communities can require decades to recover from fishing. *Mar. Ecol. Prog. Ser*. **484**, 155–171 (2013).

27. Shephard, S. *et al*. Modelling recovery of Celtic Sea demersal fish community size-structure. *Fish. Res.* **140**, 91–95 (2013).

28. Rossberg, A. G. Part-whole relations between food webs and the validity of local food-web descriptions. *Ecological Complexity* **5**, 121–131 (2008).

29. Cattin, M.-F., Bersier, L.-F., Banašek-Richter, C., Baltensperger, R. & Gabriel, J.-P. Phylogenetic constraints and adaptation explain food-web structure. *Nature* **427**, 835–839 (2004).

30. Bersier, L.-F. & Kehrli, P. The signature of phylogenetic constraints on food-web structure. *Ecological Complexity* **5**, 132–139 (2008).

31. Naisbit, R. E., Rohr, R. P., Rossberg, A. G., Kehrli, P. & Bersier, L.-F. Phylogeny versus body size as determinants of food web structure. *Proc. R. Soc. B* **279**, 3291–3297 (2012).

32. Taylor, P. J. Developmental versus morphological approaches to modelling ecological complexity. *Oikos* **55**, 434–436 (1989).

33. van Leeuwen, E., Brännström, Å., Jansen, V. A. A., Dieckmann, U. & Rossberg, A. G. A generalized functional response for predators that switch between multiple prey species. *J. Theor. Biol.* **328**, 89–98 (2013).

34. Brose, U., Williams, R. J. & Martinez, N. D. Allometric scaling enhances stability in complex food webs. *Ecol. Lett.* **9**, 1228–1236 (2006).

35. Rossberg, A. G. A complete analytic theory for structure and dynamics of populations and communities spanning wide ranges in body size. *Adv. Ecol. Res.* **46**, 429–522 (2012).

36. Metz, J. A. J. Fitness. *Evolutionary Ecology, Vol. [2] of Encyclopaedia of Ecology, 5 Vols*, 1599-1612 (Elsevier, Oxford, 2008).

37. Binzer, A. *et al*. The susceptibility of species to extinctions in model communities. *Basic Appl. Ecol.* **12**, 590–599 (2011).

38. Curtsdotter, A. *et al*. Robustness to secondary extinctions: Comparing trait-based sequential deletions in static and dynamic food webs. *Basic Appl. Ecol*. **12**, 571–580 (2011).

39. Riede, J. O. *et al*. Size-based food web characteristics govern the response to species extinctions. *Basic Appl. Ecol*. **12**, 581–589 (2011).

40. Enquist, B. J., Brown, J. H. & West, G. B. Allometric scaling of plant energetics and population density. *Nature* **395**, 163–165 (1998).

41. Mackinson, S. & Daskalov, G. *An ecosystem model of the North Sea to support an ecosystem approach to fisheries management: description and parameterization* (CEFAS, Science series: technical report No 142, 2008).

42. Robinson, L. A. *et al*. Length-weight relationships of 216 North Sea benthic invertebrates and fish. *J. Mar. Biolog. Assoc. U.K.* **90**, 95–104 (2010).

43. Drossel B., McKane A. J. & Quince C. The impact of nonlinear functional responses on the long-term evolution of food web structure. *J. Theor. Biol*. **229**, 539–548 (2004).

44. Aban, I. B., Meerschaert, M. M. & Panorska, A. K. Parameter estimation for the truncated Pareto distribution. *J. Am. Stat. Assoc.* **101**, 270–277 (2006).

45. Berlow, E. L. *et al.* Simple predictions of interaction strengths in complex food webs. *Proc. Natl. Acad. Sci. USA* **106**, 187–191 (2009).

46. Rossberg, A. G. & Farnsworth, K. D. Simplification of structured population dynamics in complex ecological communities. *Theor. Ecol.* **4**, 449–465 (2011).

47. Simon, N., Cras, A.-L., Foulon, E. & Lemée, R. Diversity and evolution of marine phytoplankton. *C. R. Biol.* **332**, 159–170 (2009).

48. Heath, M. R. Changes in the structure and function of the North Sea fish foodweb, 1973-2000, and the impacts of fishing and climate. *ICES J. Mar. Sci.* **62**, 847–868 (2005).

49. Klumpp, D. W & Polunin, N. V. C. Partitioning among grazers of food resources within damselfish territories on a coral reef. *J. Exp. Mar. Biol. Ecol.* **131**, 1–22 (1989).

50. Hendriks, A. J. Allometric scaling of rate, age and density parameters in ecological models. *Oikos* **86**, 293–310 (1999).

51. Vandermeer, J. H. The community matrix and the number of species in a community. *Am. Nat.* **104**, 73–83 (1970).

52. Wilson, W. G. *et al*. Biodiversity and species interactions: extending Lotka-Volterra community theory. *Ecol. Lett.* **6**, 944–952 (2003).

53. Wilson, W. G. & Lundberg, P. Biodiversity and the Lotka-Volterra theory of species interactions: open systems and the distribution of logarithmic densities. *Proc. R. Soc. B*

271, 1977–1984 (2004).

54. Bastolla, U., Lässig, M., Manrubia, S. C. & Valleriani, A. Biodiversity in model ecosystems, I: coexistence conditions for competing species. *J. Theor. Biol.* **235**, 521–530 (2005).

55. MacArthur, R. Species packing, and what interspecies competition minimizes. *Proc. Natl. Acad. Sci. USA* **64**, 1369–1371 (1969).

56. MacArthur, R. Species packing and competitive equilibrium for many species. *Theor. Popul. Biol.* **1**, 1–11 (1970).

57. Loreau, M. *From Populations to Ecosystems: Theoretical Foundations for a New Ecological Synthesis* (Princeton University Press, Princeton, 2010).

58. Sylvester, J. J. A demonstration of the theorem that every homogeneous quadratic polynomial is reducible by real orthogonal substitutions to the form of a sum of positive and negative squares. *Philosophical Magazine Series* **4**, 138–142 (1852).

59. Getz, W. M. Some stability results for a system of quadratic differential equations in population dynamics. *Proceedings of the Symposium on Differential Equations*. Nat. Res. Inst. Math. Sci. special report no. WISK 161 (1975).

60. Brey, T. & Clarke, A. Population dynamics of marine benthic invertebrates in Antarctic and subantarctic environments: are there unique adaptations? *Antarc. Sci.* **5**, 253–266

(1993).

61. Epstein, H. E., Walker, M. D., Chapin III, F. S. & Starfield, A. M. A transient, nutrient-based model of arctic plant community response to climatic warming. *Ecol. App.* **10**, 824–841 (2000).

62. Jennings, S., Dinmore, T. A., Duplisea, D. E., Warr, K. J. & Lancaster, J. E. Trawling disturbance can modify benthic production processes. *J. Anim. Ecol.* **70**, 459–475 (2001).
